# Dysregulation of T_FH_-B-T_RM_ lymphocyte cooperation is associated with unfavorable anti-PD-1 responses in *EGFR*-mutant lung cancer

**DOI:** 10.1038/s41467-021-26362-0

**Published:** 2021-10-18

**Authors:** Jae-Won Cho, Seyeon Park, Gamin Kim, Heonjong Han, Hyo Sup Shim, Sunhye Shin, Yong-Soo Bae, Seong Yong Park, Sang-Jun Ha, Insuk Lee, Hye Ryun Kim

**Affiliations:** 1 Department of Biotechnology, College of Life Science and Biotechnology, Yonsei University, Seoul, 03722 Korea; 2 Department of Biochemistry, College of Life Science and Biotechnology, Yonsei University, Seoul, 03722 Korea; 3 Division of Medical Oncology, Department of Internal Medicine, Yonsei Cancer Center, Yonsei University College of Medicine, Seoul, 03722 Korea; 4 Department of Pathology, Yonsei University College of Medicine, Seoul, 03722 Korea; 5 Department of Biological Sciences, Science Research Center (SRC) for Immune Research on Non-lymphoid Organ (CIRNO), Sungkyunkwan University, Jangan-gu, Suwon, Gyeonggi-do, 16419 Korea; 6 Department of Thoracic and Cardiovascular Surgery, Yonsei University College of Medicine, Seoul, 03722 Korea

**Keywords:** Cancer microenvironment, Non-small-cell lung cancer, Tumour immunology

## Abstract

Patients with non-small cell lung cancer (NSCLC) with epidermal growth factor receptor (*EGFR*) mutations exhibit an unfavorable response to PD-1 inhibitor through unclear mechanisms. Hypothesizing that *EGFR* mutations alter tumor-immune interactions, we compare tumor-infiltrating lymphocytes between EGFR mutant (EGFR-MT) and wild type (EGFR-WT) tumors through single-cell transcriptomic analysis. We find that B cells, CXCL13-producing follicular helper CD4^+^ T (T_FH_)-like cells, and tissue-resident memory CD8^+^ T (T_RM_)-like cells decreased in EGFR-MT tumors. The NOTCH-RBPJ regulatory network, which is vital for persistence of T_RM_ state, is perturbed, and the interactions between T_FH_ and B cells through the CXCL13-CXCR5 axis disappear in EGFR-MT tumors. Notably, the proportion of T_RM_-like cells is predictive for anti-PD-1 response in NSCLC. Our findings suggest that the impairment of T_FH_-B-T_RM_ cooperation in tertiary lymphoid structure formation, accompanied by the dysregulation of T_RM_ homeostasis and the loss of T_FH_-B crosstalk, underlies unfavorable anti-PD-1 response in EGFR-MT lung tumors.

## Abstract (web summary)

EGFR mutant lung tumours do not respond favourably to immunotherapy. Here, using single cell sequencing, the authors find that tissue resident memory CD8^+^ T like cells are reduced in the immune landscape of EGFR mutant tumours in comparison to wild type tumours and the presence of these cells may predict response to immunotherapy.

## Introduction

Epidermal growth factor receptor (*EGFR*) mutations are major drivers of oncogenic alterations that account for an important molecular subtype of lung adenocarcinoma, comprising 40–60% of cases in the South-East Asian population and 10–20% among Caucasian patients^1^. Patients with lung cancer with sensitizing-*EGFR* mutations clinically benefit from EGFR-tyrosine kinase inhibitors (TKIs) as first-line therapies^1–3^. However, acquired resistance inevitably develops within 9–18 months, which is the main limitation to the effectiveness of EGFR-TKIs^1–3^.

Programmed death receptor-1 (PD-1) inhibitors improve survival outcomes by offering a durable response in patients with non-small cell lung cancer (NSCLC)^4–7^. Accordingly, these inhibitors are used as first- and second-line therapies for NSCLC^4–7^. With the increase in the number of checkpoint inhibitor-based clinical trials being conducted, interest in integrating immunotherapy for patients with EGFR-mutant type (EGFR-MT) lung cancer has also increased. However, recent large-scale clinical trials and meta-analysis data have revealed unfavorable treatment outcomes of these EGFR-MT patients compared to those with EGFR wild-type (EGFR-WT), indicating that patients with EGFR-MT do not benefit from PD-1 inhibitors^8–10^. In addition, pembrolizumab for the treatment of PD-L1-positive EGFR-TKI naive patients with sensitizing-*EGFR* mutation did not show objective response benefit^11^. A recent clinical trial study reported that a combination of an anti-PD-L1 inhibitor, atezolizumab, with bevacizumab and chemotherapy showed a survival benefit in pretreated EGFR-MT patients who had failed TKI^12^. Determining the mechanisms underlying the lower efficacy of PD-1 inhibitors in patients with EGFR-MT than EGFR-WT can guide the development of treatment strategies to improve the survival outcomes of these patients.

We predicted that the TME of EGFR-MT tumors differs from that of EGFR-WT tumors in patients with NSCLC. However, previous studies using immunohistochemistry (IHC) approaches did not comprehensively compare the TMEs of the two lung cancer subtypes. Single-cell RNA sequencing (scRNA-seq) enables high-resolution dissection of cellular heterogeneity of solid tissues, including tumors. A systematic comparison of single-cell transcriptome profiles between EGFR-WT and EGFR-MT tumors can reveal immune cell subsets that differentially accumulate between groups of molecular subtypes in NSCLC tumors, some of which may provide important clues regarding their difference in clinical outcomes. Additionally, identifying biomarkers and therapeutic targets can improve anti-PD-1 immunotherapy. Although multiple scRNA-seq studies have depicted immune cell landscapes of lung cancers^13–16^, EGFR-WT and EGFR-MT tumors have not been systematically compared.

In this work, we compare the single-cell transcriptome of tumor-infiltrating immune cells between the two molecular subtypes of lung cancer and seek key cell subsets and their molecular features associated with *EGFR* mutation. We find that B cells, CXCL13-producing follicular helper CD4^+^ T (T_FH_)-like cells, and tissue-resident memory CD8^+^ T (T_RM_)-like cells are depleted in EGFR-MT compared to EGFR-WT. Homeostasis of T_RM_ cells and CXCL13–CXCR5 axis-mediated crosstalk between T_FH_ and B cells are dysregulated in EGFR-MT tumors. Finally, the proportion of cytotoxic T_RM_-like cells estimated by the expression signature reveals a highly predictive value for the anti-PD-1 response. Altogether, these results suggest the importance of T_FH_–B–T_RM_ lymphocyte cooperation in the formation of tertiary lymphoid structure (TLS) for antitumor immunity. Furthermore, its impairment in EGFR-MT tumor results in an unfavorable response to anti-PD-1 immunotherapy.

## Results

### Comparative scRNA-seq analysis reveals immune cell subsets differentially accumulated between EGFR-mutant and wild-type tumors in NSCLC

**Fig. 1:**
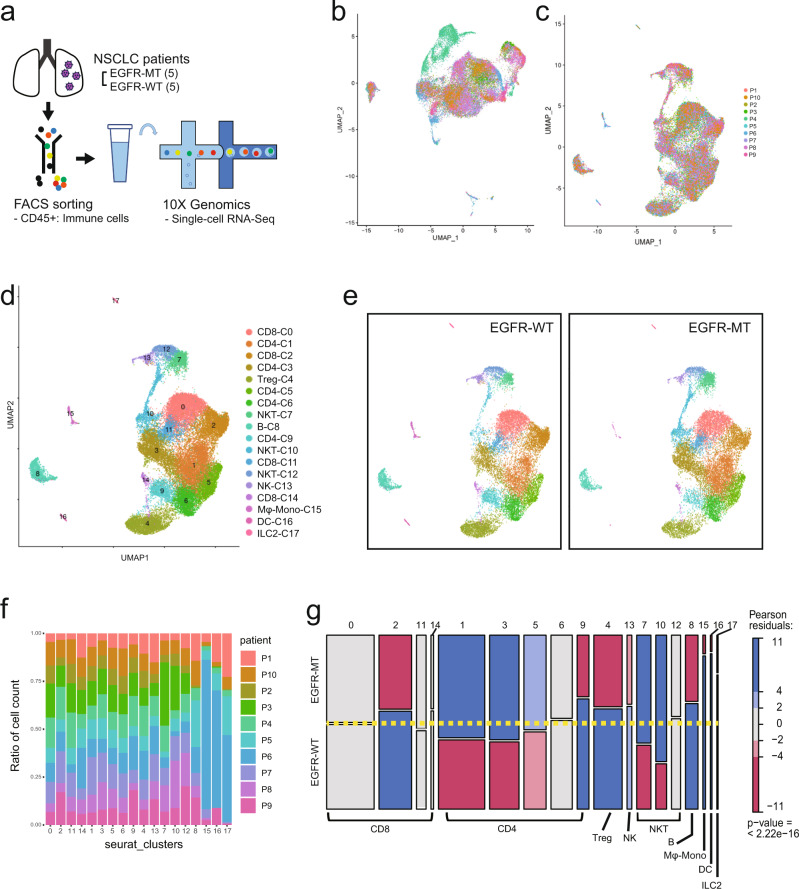
Overview of comparative scRNA-seq analysis for lung tumor samples. **a** Schematic overview of scRNA-seq analysis for lung tumor samples from 10 patients (P1–10) of mutant or wild-type EGFR. **b**, **c** UMAP visualization of scRNA-seq data before (**b**) and after (**c**) batch correction. **d** UMAP plot of 18 clusters (C0–17) of immune cells categorized into major immune cell types. The order of cluster number corresponds to the relative cell count. Clusters were annotated by canonical marker gene expression. **e** UMAP plot of 18 clusters of immune cells from either EGFR-WT (left) or EGFR-MT (right). **f** Stacked-barplot of the cell count for each patient in 18 clusters. **g** Mosaic plot of 18 clusters, each of which is composed of EGFR-WT and EGFR-MT groups. Red color indicates significantly decrease in abundance, whereas blue color indicates significantly increase in abundance (|Pearson residual| > 2 for *p*-value < 0.05, |Pearson residual| > 4 for *p*-value < 0.0001). The *p*-value shown in the mosaic plot was obtained from a one-sided chi-square test. Source data are provided as a Source Data file.

To compare the cellular landscape between two different molecular subtypes of lung cancer based on the *EGFR* mutation status, we prepared tumor specimens surgically resected from 10 patients with NSCLC in stages IA–IIIA: five EGFR-WT and five EGFR-MT (Supplementary Data 1). Focusing on the landscape of tumor-infiltrating immune cells, we sorted CD45^+^ leukocytes from tumor tissues and conducted scRNA-seq using 10X Genomics technology for individual patients (Fig. 1a and Supplementary Fig. S1). A total of 33,719 cells were sequenced, 31,887 of which passed quality control (Supplementary Data 1). We preprocessed scRNA-seq data using Seurat v.3.0 package^17^ and visualized them through UMAP dimension reduction^18^. Systematic bias was observed among data sets across different patients (Fig. 1b). To correct batch effects, scRNA-seq data were integrated using Seurat v.3.0, which is based on canonical correlation analysis and anchoring data sets between samples^17^. After batch correction, the homogeneous population structures of patients and mutation status were reduced in the latent space by UMAP dimensions (Fig. 1c). Using the Louvain algorithm of network community detection, we defined 18 clusters (C0–17) corresponding to distinct cell subpopulations (Fig. 1d). Based on the expression of canonical marker genes (Supplementary Fig. S2), we annotated clusters for major cell types such as CD8^+^ T cell (C0, C2, C11, and C14) (Supplementary Figs. S3 and 4), CD4^+^ T cell (C1, C3, C5, C6, and C9) (Supplementary Figs. S3 and 4), regulatory T cell (C4) (Supplementary Figs. S3 and 4), natural killer (NK) cell (C13) (Supplementary Fig. S5), natural killer T (NKT) cell (C7, C10, and C12) (Supplementary Fig. S5), B cell (C8) (Supplementary Fig. S6), macrophage and monocyte (C15) (Supplementary Fig. S7), dendritic cell (DC) (C16) (Supplementary Fig. S8), and innate lymphoid cell 2 (ILC2) (C17) (Supplementary Fig. S9). Many clusters apparently had unequal cell distributions between EGFR-WT and EGFR-MT (Fig. 1e), indicating differences in the composition of tumor-infiltrating immune cells between the two molecular subtypes of NSCLC. The majority of the clusters were composed of cells derived from most of the 10 patients without severe bias toward a specific patient. However, the three smallest clusters—C15 (macrophage and monocyte), C16 (DC), and C17 (ILC2)—were mostly derived from patient 6 (P6) (Fig. 1f and Supplementary Data 2). Thus, we excluded these clusters from downstream analyses to avoid formulating hypotheses based on patient-biased data.

To investigate proportional changes across the 15 cell subsets (C0–C14) between EFGR-WT and EGFR-MT, we evaluated deviations in the observed cell count from the expected counts for each subset using Pearson residual (*r*). As the sign of the residual indicates the direction of the difference in the observed count from the expected count, *r* > 0 and *r* < 0 represent augmentation and depletion compared to the expected count, respectively. We considered only subsets with *r* > 3.5 and *r* < −3.5 as augmentation and depletion with high confidence, respectively, for follow-up functional interpretation (see “Methods” section for a detailed description of *r* threshold selection). We visualized the results of the goodness of fit test using a mosaic plot, in which subsets with a deviation of observed from expected cell counts are indicated in blue (augmentation), red (depletion), or gray (no significant change) (Fig. 1g). The goodness of fit for all subsets was also evaluated by the chi-square statistic (*p*-value of Fig. 1g). We observed above-threshold level augmentation or depletion (*r* > 3.5 or *r* < −3.5) of the cell count for EGFR-WT and EGFR-MT from eight subsets (Supplementary Data 2). We expected that a successful interpretation of differentially accumulated subsets between EGFR-WT and EGFR-MT would provide clues to understanding the mechanisms underlying unfavorable anti-PD-1 response in patients with EGFR-MT NSCLC. To characterize individual subsets for each major cell type (e.g., CD8^+^ T, CD4^+^ T, Treg, NK, NKT, and B cells), we identified differentially expressed genes (DEGs) of a subset compared to all other subsets for the same major cell type (subset-DEG) and DEGs between EGFR-WT and EGFR-MT for each subset (group-DEG) (Supplementary Data 3).

### Proportion of cytotoxic NK cells decreases, whereas that of NKT cells with low cytotoxicity increases in EGFR-MT tumors

**Fig. 2:**
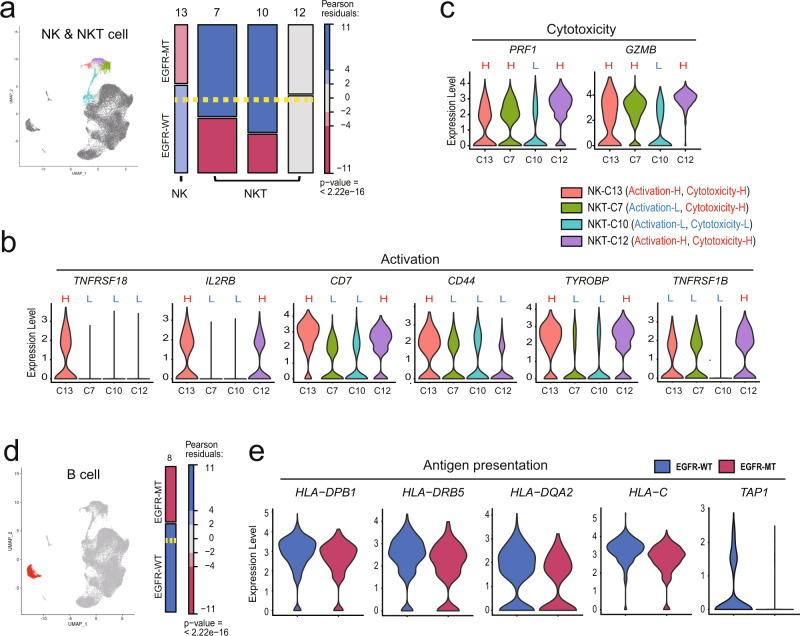
Characterization of subsets for NK, NKT, and B cells based on marker gene expression. **a** UMAP and mosaic plots for NK and NKT cells. The *p*-value shown in the mosaic plot was obtained from a one-sided Chi-square test. **b**, **c** Violin plots for the expression of genes involved in activation (**b**) and cytotoxicity (**c**) of NK or NKT lymphocytes across subsets (H, high expression level; L, low expression level). Each subset was characterized based on the expression levels of genes involved in activation and cytotoxicity. For example, NKT-C7 was characterized by low activation and high cytotoxicity. **d** UMAP and mosaic plots for B cells. **e** Violin plots for the expression of genes involved in antigen presentation and processing in B cells of EGFR-WT or EGFR-MT tumor.

NK and NKT cells are cytotoxic lymphocytes of the innate immune system that provide the first line of defense in the body before activating the adaptive immune system. Although these cells have a limited range of antigen specificity and lower capacity for memory-generation, they can kill cancer cells directly via cytotoxic molecules^19^. The subset of cytotoxic NK cells (C13) was depleted in EGFR-MT, although its significance was not above the pre-determined threshold (*r* = −3.34) (Fig. 2a). Among the three subsets for NKT cells (C7, C10, and C12), the proportion of NKT-C7 and NKT-C10 was significantly augmented in EGFR-MT (*r* = 6.35 and 10.57, respectively; Fig. 2a). The subset-DEG analysis revealed that NK-C13 and NTK-C12, which were depleted and unchanged in EGFR-MT, respectively, expressed more genes involved in activation (*TNFRSF18*, *IL2RB*, *CD7*, *CD44*, *TYROBP*, and *TNFRSF1B*)^20–22^ (Fig. 2b) and cytotoxicity (*PRF1* and *GZMB*)^23^ (Fig. 2c) than other subsets. In contrast, NKT-C7 and NKT-C10 proportion increased in EGFR-MT and these subsets expressed genes involved in either activation or cytotoxicity at lower levels than in other subsets. Overall, NKT-C10 is likely to have the lowest cytotoxic activity among all NK and NKT cell subsets, as the expression of genes involved in activation and cytotoxicity was lower than that in all other subsets (Fig. 2b, c), which is consistent with the highest augmentation in EGFR-MT (*r* = 10.57) (Fig. 2a). In summary, single-cell transcriptome analysis revealed the augmentation of NKT cell subsets with relatively low expression of genes involved in activation and cytotoxicity in EGFR-MT.

### Proportion of B cells decreases with reduced antigen presentation in EGFR-MT tumors

B cells secrete antibodies to neutralize antigens or tag tumor cells for attack by other immune cells^24^. Recently, tumor-infiltrated B cells have been reported to promote the response to immunotherapy and survival in cancer patients through the formation of TLSs, in which B cells can present a tumor-derived neoantigen to T cells to activate them^25–28^. We observed a significant depletion of B cells in EGFR-MT compared to EGFR-WT (*r* = −6.23, Fig. 2d), suggesting that EGFR-MT tumor has a lower proportion of tumor-infiltrating B cells than EGFR-WT. In addition, we observed that B cells from EGFR-MT showed lower expression levels of HLA genes (MHC class I: *HLA-C* and MHC class II: *HLA-DPB1*, *HLA-DRB5*, and *HLA-DQA2*) and *TAP1* (antigen peptide transporter involved in processing antigens that will be presented to MHC class I) (Fig. 2e). These results suggest that B cells in EGFR-MT tend to less effectively activate both CD8^+^ and CD4^+^ T cells than EGFR-WT, due to the lower expression of MHC class I and II, respectively.

### Proportion of CXCL13-producing T_FH_-like cells decreases, whereas that of immunosuppressive CD4^+^ T cells increases in EGFR-MT tumors

**Fig. 3:**
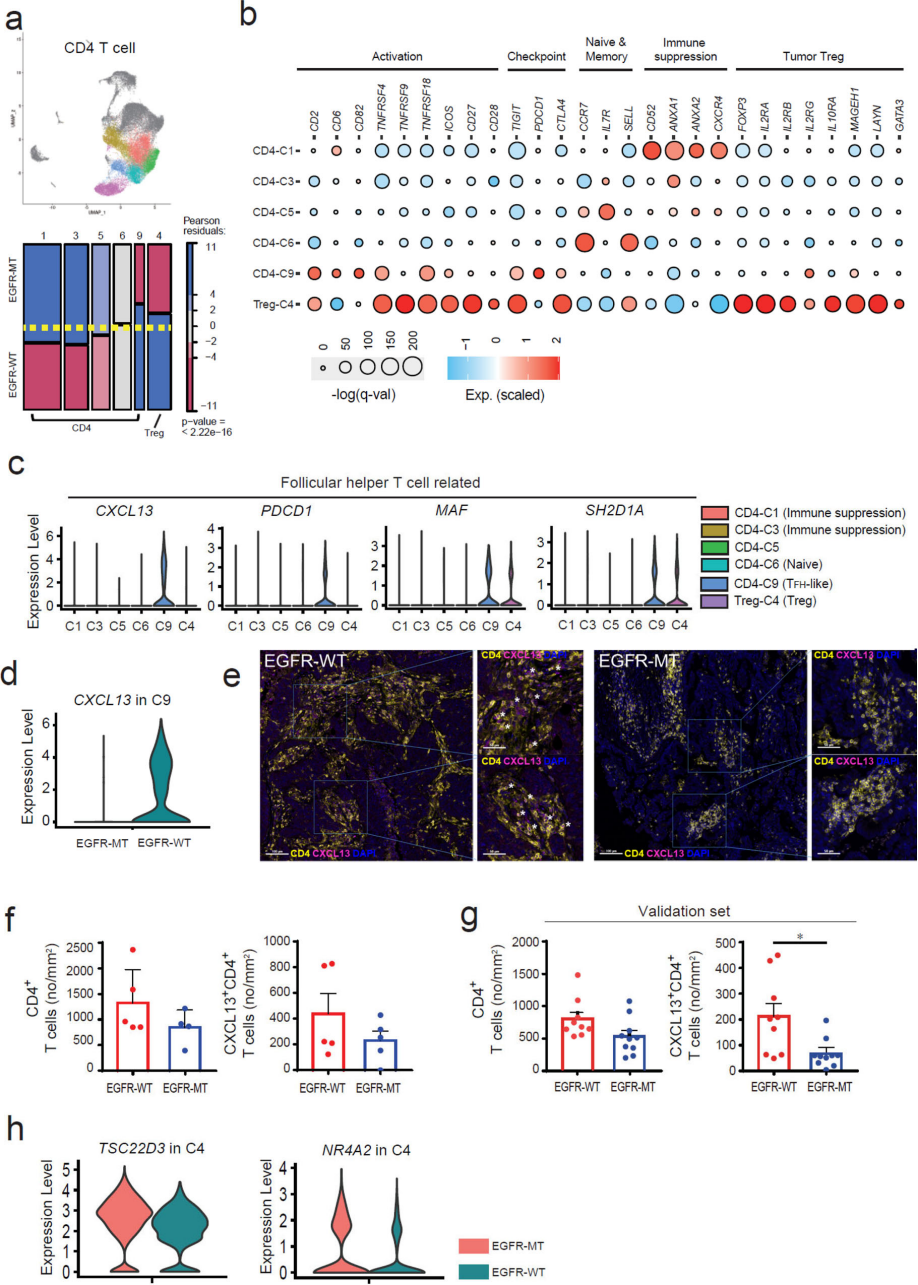
Characterization of subsets for CD4+ T cells based on marker gene expression and multiplexed immunofluorescence (IF) analysis. **a** UMAP and mosaic plot for CD4^+^ T cells. The *p*-value shown in the mosaic plot was obtained from a one-sided chi-square test. **b** Dot plots for the expression of marker genes for various functional properties of CD4^+^ T cells. Subset-DEGs are represented as a red circle with statistical significance indicated by the dot size. **c** Violin plots for the expression of genes related to follicular helper T cells across subsets. **d** Violin plots for the expression of *CXCL13* genes in C9 cells of EGFR-WT and EGFR-MT. **e** Representative multiplexed IF (scale bar 100 μm: left, scale bars 50 μm: right magnified) of CD4 and CXCL13 in tumor specimens from EGFR-WT (*n* = 14) and EGFR-MT (*n* = 14) patients. Asterisks mark the double-positive CD4^+^CXCL13^+^ cells. **f** The cell counts of CD4^+^, CXCL13^+^CD4^+^ T cells per mm^2^ of EGFR-WT (*n* = 5) and EGFR-MT (*n* = 4) tumors were depicted in the bar graph. **g** The cell counts of CD4^+^, CXCL13^+^CD4^+^ T cells per mm^2^ of tumors from independent patients with EGFR-WT (*n* = 9) and EGFR-MT (*n* = 10) for validation were depicted in the bar graph (*p ≤ 0.05, two-sided test). **h** Violin plots for the expression of *TSC22D3* and *NR4A2*, which are the genes involved in Treg activation, in C4 cells of EGFR-WT and EGFR-MT. Data are represented as mean ± SEM. Source data are provided as a Source Data file.

Along with CD8^+^ T cells, CD4^+^ T cells are major lymphocytes governing adaptive immunity; they differentiate into diverse types of helper T cells that activate other immune cells, including cytotoxic T cells. Accordingly, various conventional CD4^+^ T cells are involved in antitumor or tumor-promoting processes, whereas CD4^+^ Treg suppresses antitumor immunity in the TME. We obtained six subsets of CD4^+^ T cells (Fig. 3a), which were characterized by subset-DEG analysis for various categories of immune signature genes (Fig. 3b). Among the six CD4^+^ subsets, the two largest ones showed significant augmentation in EGFR-MT (C1 and C3 by *r* = 8.48 and 7.66, respectively). We found that these subsets showed a high expression of genes involved in CD4^+^ T cell-mediated immune suppression (*CD52*^29,30^, *ANXA1*^31,32^, *ANXA2*^33^, and *CXCR4*^34^). The C5 subset, for which the marker-based cellular identity was relatively unclear, showed augmentation in EGFR-MT with a subthreshold level of significance (*r* = 2.74). The C6 subset, annotated as naive CD4^+^ T cells based on the high expression of *CCR7* and *SELL*, showed no significant difference in relative abundance between EGFR-WT and EGFR-MT.

Notably, the C9 subset was CD4^+^ T cells with the largest depletion in EGFR-MT (*r* = −7.37). We found several genes related to T_FH_ cells (*CXCL13*^35^, *ICOS*^36^, *MAF*^37^, *PDCD1*^35^, and *SH2D1A*^35^) among the subset-DEGs for C9 (Fig. 3b, c and Supplementary Data 3). T_FH_ cells reportedly help B cells by promoting the maturation of antibody affinity in germinal centers (GC)^35^. PD-1 regulates GC positioning and helps with the function of T_FH_ cells^38^. Human T_FH_ cells produce CXCL13, a major chemoattractant in homing B cells to follicles^35^. CXCL13 has been associated with TLS development^39,40^. CXCR5, a receptor for CXCL13 and another hallmark of T_FH_ cells, was not detected as a subset-DEG of C9. Moreover, the C9 subset does not express *BCL6*, a lineage-defining transcription factor of T_FH_ cells^35^. These CXCR5 and BCL6-negative T_FH_-like cells that produce CXCL13 are found in local inflammatory sites^41^, where TLS development frequently occurs^42^. Given that the major role of the T_FH_ cells is to recruit B cells, depletion of CXCL13-producing T_FH_-like cells in EGFR-MT may result in the inefficient formation of TLS. The observed depletion of tumor-infiltrating B cells (Fig. 2d) also suggests lower TLS development in EGFR-MT. Recently, B cells and TLS have been shown to be associated with responses to immunotherapy^26–28^. We also observed a substantially lower level of *CXCL13* expression in EGFR-MT than in EGFR-WT tumor (Fig. 3d), which further supports the hypothesis that CXCL13-producing T_FH_-like cells contribute to the anti-PD-1 response through recruiting B cells to functional TLS in tumors. To confirm the reduced abundance of CXCL13^+^CD4^+^ T cells in EGFR-MT compared with EGFR-WT tumor, inferred from the single-cell transcriptome analysis, we performed the multiplexed immunofluorescence (IF) staining in archival tissue specimens of the same samples. We could obtain results of multiplexed IF for nine of the prior samples, because the sample quantity for the P3 patient was not sufficient for the downstream analysis. The results of multiplexed IF confirmed the reduced number of CD4^+^ and CXCL13^+^CD4^+^ T cells in EGFR-MT compared with EGFR-WT tumors (Fig. 3e, f and Supplementary Figs. S10, S11, Supplementary Data 4a). Although not statistically significant due to the small sample size, these results are congruous with the observations from the single-cell transcriptional analysis. To validate these findings further, we performed a similar analysis on a validation set of tumor samples from 19 independent NSCLC patients with EGFR-WT (*n* = 9) or EGFR-MT (*n* = 10) (Supplementary Data 5a). For this validation set with a larger sample size, we could observe significantly reduced frequencies of CXCL13^+^CD4^+^ T cells in EGFR-MT compared with EGFR-WT tumors for the validation set (Fig. 3g and Supplementary Fig. S12, Supplementary Data 4c).

The C4 subset was annotated as Treg cells based on the high expression signature genes of tumor-infiltrating Tregs (*FOXP3*, *IL2RA*, *IL2RB*, *IL2RG*, *IL10RA*, *MAGEH1*, *LAYN*, and *GATA3*). They also showed a high expression of genes involved in T cell activation (*CD27*, *CD28*, *ICOS*, and *TNFRSF4* encoding OX40, *TNFRSF9* encoding 41BB, and *TNFRSF18* encoding GITR) and checkpoint molecules (*TIGIT* and *CTLA4*). Counterintuitively, we observed a depletion of the Treg subset in EGFR-MT (Fig. 3a). However, we also found that Treg cells of EGFR-MT tumors expressed genes known to promote Treg activity (*TSC22D3* encoding GILZ^43^ and *NR4A2*^44^) at levels significantly higher than those in EGFR-WT (Fig. 3h and Supplementary Data 3), which may compensate for its disadvantages in abundance.

### Proportion of cytotoxic CD8^+^ T_RM_-like cells decreases in EGFR-MT tumors

**Fig. 4:**
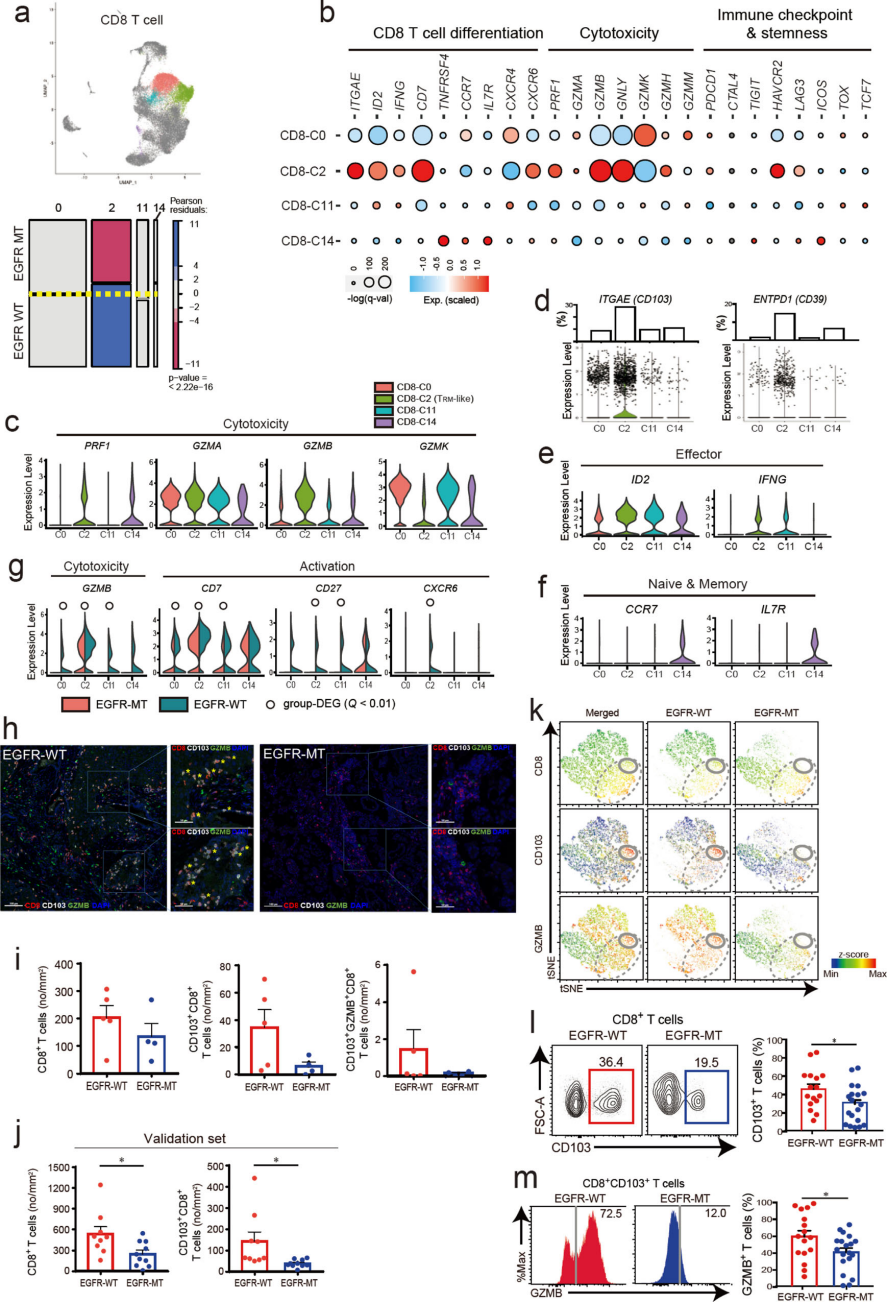
Characterization of subsets for CD8+ T cells based on marker gene expression, multiplexed immunofluorescence (IF), and flow cytometry analysis. **a** UMAP and mosaic plot for CD8^+^ T cells. The *p*-value shown in the mosaic plot was obtained from a one-sided chi-square test. **b** Dot plots for the expression of marker genes affecting various functional properties of CD8^+^ T cells. Subset-DEGs are indicated as red circles, with statistical significance indicated by the size of the dot. **c** Violin plots for the expression of genes involved in cytotoxicity. **d** Violin plots for the expression of hallmark genes for tissue-residency (*ITGAE*) and tumor reactivity (*ENTPD1*) of CD8^+^ T cell subset. The expression levels for individual cells are indicated along with the violin plot, and the proportion (%) of cells that express each hallmark gene is also presented. **e** Violin plots for the expression of genes related to effector function. **f** Violin plots for the expression of genes related to the naive or memory state of T cells. **g** Violin plots for the expression of genes involved in cytotoxicity and T cell activation. Violin plots for two groups (EGFR-WT and EGFR-MT) are fused to facilitate comparison. DEGs with a higher expression for EGFR-WT are indicated as group-DEG. **h** Representative multiplexed IF of CD8, CD103, and GZMB in tumor specimens from EGFR-WT (*n* = 14) (scale bar 100 μm: left, scale bars 50 μm: right magnified) and EGFR-MT (*n* = 14) (scale bar 100 μm: left, scale bars 50 μm: right magnified) patients. Asterisks mark the triple-positive CD8^+^CD103^+^GZMB^+^ cells. **i** The cell counts of CD8^+^, CD8^+^CD103^+^, and CD8^+^CD103^+^GZMB^+^ per mm^2^ in EGFR-WT (*n* = 5) and EGFR-MT (*n* = 4) patients are depicted in the bar graph. **j** The cell counts of CD8^+^ and CD8^+^CD103^+^ per mm^2^ tumors collected from independent patients with EGFR-WT (*n* = 9) and EGFR-MT (*n* = 10) for validation were presented in the bar graph (*p ≤ 0.05, two-sided test). **k** tSNE visualization of flow cytometry data from 7000 randomly selected live and singlet-gated CD3^+^ cells isolated from EGFR-WT (*n* = 3) and EGFR-MT (*n* = 3) tumor samples. Each cell is marked by a dot whose color scales according to the *z*-score expression value of the indicated protein (CD8, CD103, or GZMB). **l** Representative contour plot and summarized graph for the expression of CD103^+^ cells among the CD8^+^ T cells in EGFR-WT (*n* = 16) and EGFR-MT (*n* = 19) tumors collected from independent patients for validation (*p ≤ 0.05, two-sided test). **m** Representative histogram and summarized graph for the expression of GZMB^+^ cells among CD8^+^CD103^+^ T cells in EGFR-WT (*n* = 16) and EGFR-MT (*n* = 19) tumors collected from independent patients for validation (*p ≤ 0.05, two-sided test). Data are represented as mean ± SEM. Source data are provided as a Source Data file.

CD8^+^ T cells were clustered into four different subsets (C0, C2, C11, and C14), among which, only C2 exhibited significant differences in its proportion between EGFR-WT and EGFR-MT, particularly depletion in EGFR-MT (*r* = −6.44) (Fig. 4a). We characterized each subset of CD8^+^ T cells based on the expression of various categories of signature genes (Fig. 4b) and found that the C2 subset had the highest cytotoxicity and expressed *ITGAE* (also known as *CD103*), a hallmark of T_RM_ cells^45^. In addition, the C2 subset showed a significantly higher effector signature (*ID2* and *IFNG*) and memory signature (*CD7*). T_RM_ cells are a subset of effector memory T cells in the peripheral tissue, and their roles in antitumor immunity have recently gained considerable attention^46,47^. We also found that the C2 subset highly expressed the signature genes for tumor-infiltrating T_RM_-like CD8^+^ T cells such as *PDCD1, HAVCR2,* and *LAG3*, but rarely expressed *TCF7*, a lung T_RM_ suppressor^48–50^ (Fig. 4b). Previously, *HAVCR2* (also known as *TIM-3*) expression has been reported as a feature of lung tumor T_RM_ cells that are not involved in exhaustion but rather reflect a functional state^49^. In this regard, we defined the C2 subset as T_RM_-like cells. As a fast-reactive memory T cell subset, T_RM_ cells constitutively express perforin (*PRF1*), granzyme B (*GZMB*), and IFN-gamma (*IFNG*). We found that the tumor-infiltrating T_RM_-like subset expressed a high level of *GZMB*, whereas other subsets showed much lower expression, indicating the T_RM_-like subset as a major cytolytic lymphocyte in the tumor (Fig. 4c). High expression of *ENTPD1* (also known as *CD39*) indicates that the C2 subset is composed of tumor-reactive T_RM_-like cells rather than bystander T cells^51^ (Fig. 4d). The expression of effector molecules and markers for naive/memory states suggest that C0 and C11 are effector CD8^+^ T cell subsets with lower cytotoxicity, and C14 is a naive T cell subset (Fig. 4e, f). Notably, we observed a higher expression of *GZMB* and genes involved in T cell activation in T_RM_-like cells from EGFR-WT than in those from EGFR-MT (Fig. 4g), indicating that T_RM_-like cells in EGFR-WT tumors have the highest antitumor activity for the direct killing of cancer cells.

We evaluated the protein expression of selected molecules (CD8, CD103, and GZMB) using multiplexed IF for the whole slides to confirm the presence of T_RM_-like cells in an archival tissue specimen. The abundance of CD8^+^, CD103^+^CD8^+^, and GZMB^+^ CD103^+^CD8^+^ T cells tended to be lower in EGFR-MT than EGFR-WT (Fig. 4h, i and Supplementary Figs. S10, 11, Supplementary Data 4a), consistent with the results based on the transcriptional level. For the validation set of tumor samples from 19 independent NSCLC patients (Supplementary Data 5a), we could observe a significantly lower abundance of CD8^+^ and CD103^+^CD8^+^ T cells in EGFR-MT compared with EGFR-WT (Fig. 4j and Supplementary Fig. S12, Supplementary Data 4c). For further validation, we performed a flow cytometry analysis using multiple antibodies, including anti-CD8, anti-CD103, and anti-GZMB for another validation set that consist of tumors from independent 35 NSCLC patients with EGFR-WT (*n* = 16) or EGFR-MT (*n* = 19) (Supplementary Data 5b). The T_RM_-like cell subset was highly enriched for cells with a high expression of both CD103 and GZMB (area within the solid circle) among CD8^+^ cells (area within the dotted circle) in EGFR-WT compared to EGFR-MT (Fig. 4k). The proportion of CD103^+^ cells among CD8^+^ cells and that of GZMB^+^ cells among CD8^+^CD103^+^ cells was significantly lower in EGFR-MT than EGFR-WT (Fig. 4l, m). Therefore, the depletion of CD8^+^ T_RM_-like cells in EGFR-MT lung tumors was confirmed by protein-based immunophenotyping analysis.

### Homeostasis of T_RM_-like cells is impaired in EGFR-MT tumors

Hitherto our comparative single-cell transcriptome analysis between EGFR-WT and EGFR-MT revealed three lymphocyte subsets significantly depleted in lung tumors with *EGFR* mutations: B cells, CD4^+^ T_FH_-like cells, and CD8^+^ T_RM_-like cells. Given that patients with lung cancer with *EGFR* mutations exhibit unfavorable anti-PD-1 responses, regulation of cooperation among these lymphocyte subsets may be important for antitumor immunity in lung tumors. Based on the constitutively high expression of major cytolytic enzymes, such as perforin and GZMB, in T_RM_-like cells, the persistence of T_RM_ cell state in a tumor may be a major determinant of the effective killing of cancer cells.

**Fig. 5:**
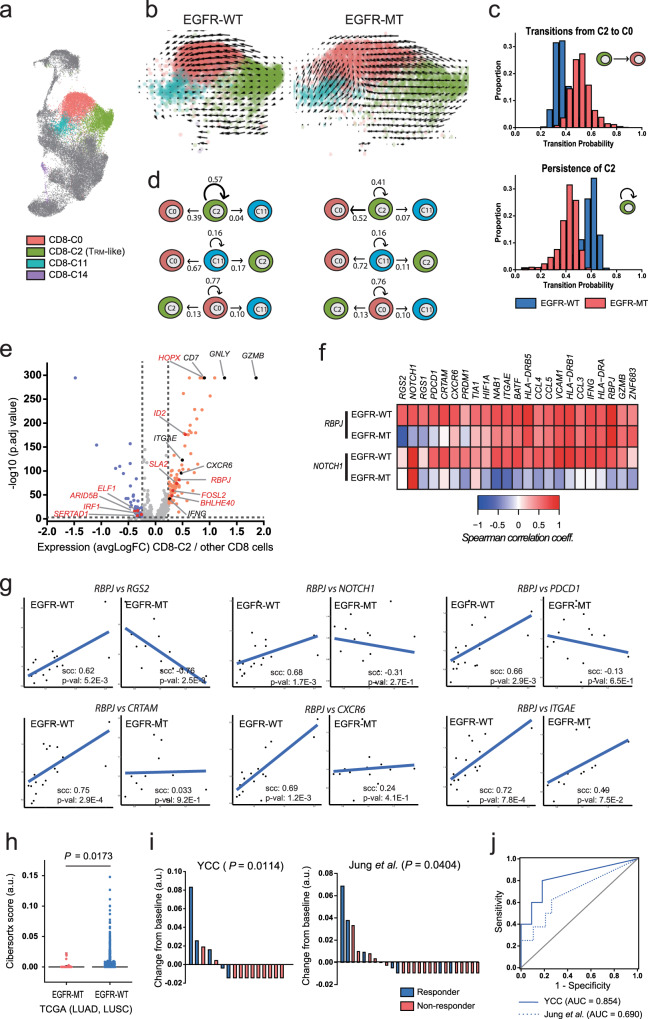
Regulation of TRM-like subset homeostasis that can predict anti-PD-1 responses in lung cancer. **a** UMAP plot for CD8^+^ T cells with color codes for four distinct cell subsets. **b** Transition probability of cells is visualized on the predefined UMAP plots for CD8^+^ T cells of EGFR-WT and EGFR-MT tumors. Only three major subsets of CD8^+^ T cells (C0, C2, and C11) are presented here. A longer arrow indicates a higher transition probability. **c** Histograms for the distribution of transition probabilities from C2 subset to C0 subset (upper) and those from C2 subset to C2 subset (i.e., C2 persistence) in two different tumor groups. From these histograms, C2 cells of EGFR-MT are more likely to transit to C0, whereas those of EGFR-WT tend to persist in the C2 state. **d** Summary of transitions among the three major subsets. C0 and C11 showed no difference in persistence between EGFR-WT and EGFR-MT (middle and bottom), whereas C2 showed higher persistence in EGFR-WT than in EGFR-MT (top). **e** Volcano plot showing DEGs in T_RM_-like cells (C2) compared to other subsets of CD8^+^ T cells. TFs are indicated by red color. **f** Heat map visualization of Spearman’s rank correlation of *RBPJ* or *NOTCH1* with genes related to T_RM_ cell differentiation or maintenance (columns) in EGFR-WT and EGFR-MT tumors. **g** Scatter plots with regression curves that highlight largely decreased or even inversed correlation between *RBPJ* and potential regulatory target genes in EGFR-MT compared with EGFR-WT tumor. **h** Boxplot for the abundance of T_RM_-like cells in EGFR-WT and EGFR-MT of TCGA LUAD and LUSC samples estimated by CIBERSORTx signature matrix score. **i** Waterfall plot to depict anti-PD-1 response based on the cohorts of patients with NSCLC recruited from Yonsei Cancer Center (YCC) and patients with NSCLC from Jung et al. The baseline represents the median level of CIBERSORTx score representing the proportion of the tumor-infiltrating T_RM_-like cells. All *p*-values were calculated using the one-tailed Mann–Whitney *U* test (testing the association of responder status with the proportion of tumor-infiltrating T_RM_-like cells). **j** Area under the receiver operating characteristics curve for retrieving responders based on the proportion of tumor-infiltrating T_RM_-like cells. Source data are provided as a Source Data file.

To evaluate the homeostasis of T_RM_-like cells in EGFR-WT and EGFR-MT tumors, we measured the transition probability between CD8^+^ subsets (C0, C2, C11, and C14; Fig. 5a) based on RNA velocity analysis using velocyto^52^, which can be estimated from the ratio of unspliced to spliced mRNA. Based on the probability of future mRNA abundance for all genes, we can predict the future state (subset) of each cell (Fig. 5b). For example, the mean transition probability from C2 to C0 was higher in EGFR-MT than in EGFR-WT, whereas that from C2 to C2 (that is, the persistence of C2 state) was higher in EGFR-WT than in EGFR-MT (Fig. 5c). We compared and calculated the transition probability among four subsets, C0, C2, C11, and C14, between EGFR-WT and EGFR-MT tumors to identify the changes in homeostasis. Notably, C2 (T_RM_-like cells) derived from EGFR-WT and EGFR-MT showed probabilities of 0.57 and 0.41 for persistence, respectively (Fig. 5d and Supplementary Data 6). In contrast, all other subsets showed similar probabilities for persistence between EGFR-WT and EGFR-MT: 0.77 vs. 0.76 for C0, 0.16 vs. 0.16 for C11, and 0.042 vs. 0.034 for C14. We also performed scVelo^53^ analysis that enables estimation of RNA velocity without steady-state assumption and observed consistent results (Supplementary Fig. S13). These results suggest that the persistence of the T_RM_ cell state is impaired in EGFR-MT lung tumors.

### NOTCH-RBPJ regulatory network for maintaining T_RM_ cells is perturbed in EGFR-MT tumors

Transcriptional regulatory networks tightly control differentiation and functional maintenance of immune cells. We hypothesized that the dysregulation of the transcriptional regulatory network for homeostasis in the T_RM_ cell state occurs in EGFR-MT lung tumors. To reconstruct the key regulatory network, we first identified differentially upregulated transcription factors (TFs) in the C2 subset compared to other CD8^+^ cell subsets (Fig. 5e). Among the upregulated TFs, *ID2*, *RBPJ*, and *BHLHE40* are known to be involved in T_RM_ cell development and homeostasis^54–56^. Particularly, RBPJ is associated with NOTCH to form a transcriptional activator required for maintaining T_RM_ cells in humans^55^. Next, we compared the co-regulatory network of RBPJ between EGFR-WT and EGFR-MT to identify the gain or loss of regulatory targets for RBPJ between the two molecular subtypes of lung tumors. To improve the robustness of gene-gene correlation analysis for highly sparse scRNA-seq data, we employed the MetaCell^57^ analysis method, which partitions single-cell transcriptome profiles into disjoint and homogeneous groups of profiles. As expected, we observed a high correlation of expression between *RBPJ* and many known genes for T_RM_ cell differentiation and maintenance^45–47^ in C2 subset cells from EGFR-WT tumors (Fig. 5f). Additionally, *NOTCH1* showed a high correlation with the same regulatory target genes, although it was not significantly upregulated in the C2 subset compared with other CD8^+^ cell subsets. Interestingly, many transcriptional correlations with *RBPJ* disappeared in the C2 subset cells of EGFR-MT. Particularly, the expression of *RGS2*, *NOTCH1, RGS1*, *PDCD1*, *CRTAM*, *CXCR6*, *TIA1*, *HIF1A*, *NAB1*, and *ITGAE* positively correlated with *RBPJ* in the C2 subset of EGFR-WT, but the gene-gene correlations were lost or even inversely correlated in the C2 subset of EGFR-MT (Fig. 5g and Supplementary Data 7). The loss of the transcriptional correlation in EGFR-MT was more marked with *NOTCH1* (Fig. 5f and Supplementary Data 7). These results indicate that the persistence of the tumor-infiltrating T_RM_ cell state depends on the NOTCH-RBPJ regulatory network, which is dysregulated in EGFR-MT lung tumors.

### Proportion of tumor-infiltrating T_RM_-like cells is highly predictive for anti-PD-1 response in NSCLC

Given that the proportion of tumor-infiltrating T_RM_-like cells decrease in EGFR-MT lung tumors, which tends to show poor response to anti-PD-1 immunotherapy, we hypothesized that the relative abundance of T_RM_-like cells in the tumor by itself could predict the anti-PD-1 response. The current gold-standard estimation of the proportion of each immune cell type is flow cytometry analysis using canonical markers specific for each cell type. However, this approach is valid for only major cell types or subtypes with well-characterized marker genes. Single-cell biology enables discovering an increasing number of cell subsets, and identifying specific markers for the subsets would be a daunting task. Therefore, quantifying cell subsets from a heterogeneous cell population using cell-type-specific expression signature has recently been employed. To infer the expression signature of the 18 subsets of immune cells from lung tumor samples, we applied CIBERSORTx^58^, a deconvolution method developed for inferring gene expression signature for a specific cell type, to the single-cell transcriptome profiles and generated a signature matrix of 18 subsets (Supplementary Data 8a). We then estimated the relative abundance of T_RM_-like cells in The Cancer Genome Atlas (TCGA) cohort of lung adenocarcinoma (LUAD) and lung squamous cell carcinoma (LUSC)^59^. Consistent with the single-cell transcriptome results, the signature score for T_RM_-like cells was significantly higher in TCGA tumors with wild-type EGFR than in the mutant type (*p* = 0.0173, one-tailed Mann–Whitney *U* test) (Fig. 5h and Supplementary Data 8b). This result confirms that the inferred expression signature is a suitable estimator of the relative abundance of T_RM_-like cells in the tumor.

Next, we evaluated the expression signature of T_RM_-like cells to predict the response to anti-PD-1 immunotherapy using bulk RNA-seq data and clinical information available for two independent public cohorts of patients with NSCLC (GSE126045 and GSE135222)^60,61^. Notably, we observed a positive correlation between the signature score, indicating an abundance of tumor-infiltrating T_RM_-like cells and anti-PD-1 response in both cohorts (Fig. 5i and Supplementary Data 8c, d). In the two NSCLC cohorts, the signature score of responders was significantly higher than that of non-responders (*p* = 0.0114 and 0.0404, one-tailed Mann–Whitney *U* test). Furthermore, we prioritized patients exhibiting the highest signature score. This stratification resulted in a large area under the receiver operating characteristic curve (AUROC) scores, indicating that a high abundance of tumor-infiltrating T_RM_-like cells is a predictive marker of the anti-PD-1 response (Fig. 5j). Notably, only 2 of the 18 patients in the cohort recruited by Yonsei Cancer Center (YCC)^60^ were EGFR-MT; one was a responder, whereas the other was not (EGFR mutation status information was not available for the cohort by Jung et al.^61^). Therefore, the identified expression signature for T_RM_-like cells by itself can be used as a biomarker to stratify patients with lung cancer for anti-PD-1 immunotherapy regardless of their *EGFR* mutation status.

### CXCL13–CXCR5-mediated T_FH_–B and T_RM_–B interactions are dysregulated in EGFR-MT tumors

**Fig. 6:**
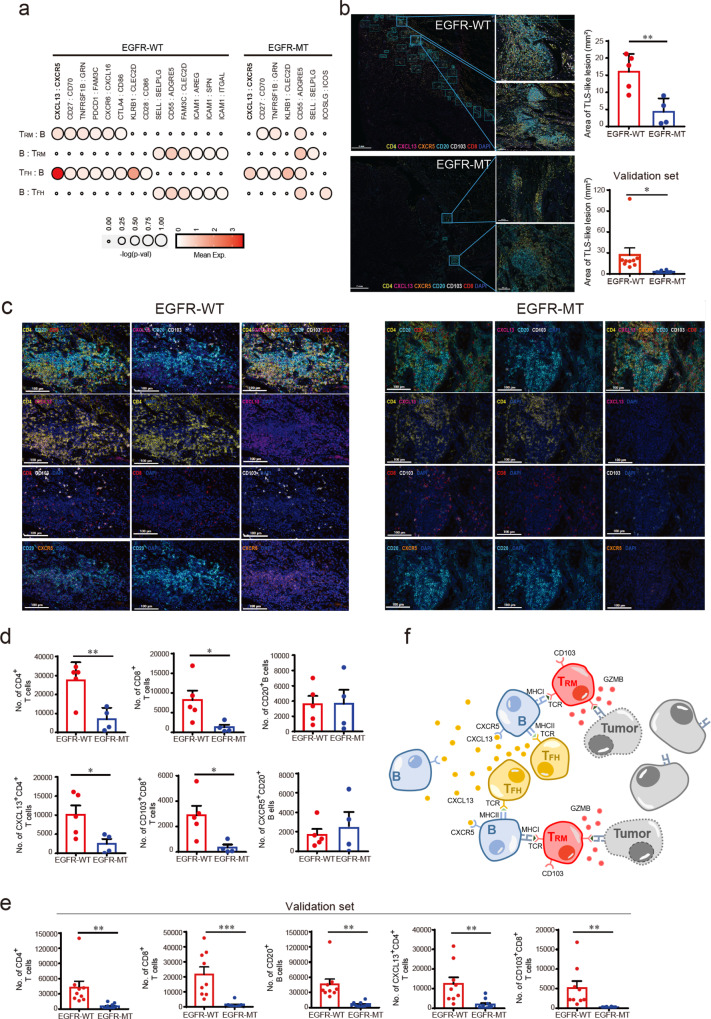
T_FH_–B–T_RM_ lymphocyte cooperation and their co-localization in lung tumors. **a** The dot plot summarizing the cell–cell interactions among CD8-C2 (T_RM_-like), B, and CD4-C9 (T_FH_-like) cells by CellPhoneDB analysis. Only significant ligand–receptor interactions are presented here. The size of the circle indicates the statistical significance, and the color gradient indicates the mean expression value of gene pairs. **b** Representative multiplexed immunofluorescence (IF) of TLS-like lesion in EGFR-WT (*n* = 14) (upper) (scale bar 100 μm: left, scale bars 50 μm: right magnified) and EGFR-MT (*n* = 14) (lower) (scale bar 100 μm: left, scale bars 50 μm: right magnified) tumors. Summarized graph for the area of TLS-like lesions in EGFR-WT (*n* = 5) and EGFR-MT (*n* = 4) tumors for single-cell analysis (**p ≤ 0.005, two-sided test) (upper panel) and in EGFR-WT (*n* = 9) and EGFR-MT (*n* = 10) tumors from independent patients for validation (*p ≤ 0.05, two-sided test) (lower panel). **c** Representative multiplexed IF of CD4, CXCL13, CD103, CD8, CXCR5, and CD20 in tumor specimens from EGFR-WT (*n* = 14) (left) (scale bar 100 μm) and EGFR-MT (*n* = 14) (right) (scale bar 100 μm) patients. **d** Summarized graph for the numbers of each cell type in total TLS-like lesions in EGFR-WT (*n* = 5) and EGFR-MT (*n* = 4) patients. The number of CD4^+^, CXCL13^+^CD4^+^ T cells, CD8^+^ T cells, CD103^+^ CD8^+^ T cells are significantly higher in EGFR-WT than EGFR-MT (*p ≤ 0.05, **p ≤ 0.005, two-sided test). **e** Summarized graph for the numbers of each cell type in total TLS-like lesions in EGFR-WT (*n* = 9) and EGFR-MT (*n* = 10) tumors from independent patients for validation. Consistently, the number of CD4^+^, CXCL13^+^CD4^+^ T cells, CD8^+^ T cells, CD103^+^ CD8^+^ T cells were significantly higher in EGFR-WT than EGFR-MT (**p ≤ 0.005, ***p ≤ 0.0005, two-sided test). **f** An illustrative summary of the proposed model for T_FH_–B–T_RM_ lymphocyte cooperation in TLS for antitumor immunity in lung cancer. Data are represented as mean ± SEM. Source data are provided as a Source Data file.

Previous studies also demonstrated that T_FH_ cells release CXCL13 to recruit B cells into TLS^35^, which then present tumor antigens to T cells^62^. As demonstrated in our study, the abundance of CXCL13-producing T_FH_-like cells was higher in EGFR-WT than in EGFR-MT patients (Fig. 3e, f). These observations suggest that recruiting B cells into the TLS of the tumor become less effective in EGFR-MT, likely due to a loss of CXCL13–CXCR5-mediated interactions, resulting in reduced cytotoxic T cell activation. To test this hypothesis, we evaluated cell-to-cell interactions among T_FH_-like cells (C9), B cells (C8), and cytotoxic T_RM_-like cells (C2) using CellPhoneDB^63^. We found more significant cell–cell interactions in EGFR-WT than EGFR-MT (Fig. 6a). We found that T_FH_–B cell interaction through the CXCL13–CXCR5 axis was substantially weaker in EGFR-MT (lower mean expression of ligand and receptor) than in EGFR-WT. Notably, we could also detect CXCL13–CXCR5-mediated interactions between T_RM_–B cells in EGFR-WT but not in EGFR-MT. Indeed, T_RM_-like cells also express *CXCL13*, albeit not as much as in T_FH_-like cells.

Recently, a PD-1^+^CD8^+^ T cell subset that secretes CXCL13 has been detected close to the B cell area of the TLS in lung cancer^64^. B cells of EGFR-WT appeared to present more molecules involved in antigen processing and presentation than EGFR-MT (Fig. 3d). These molecules will promote the activation of CD4^+^ T_FH_-like cells (through MHC class II) and CD8^+^ T_RM_ cells (through MHC class I). Subsequently, the enhanced antigen-presenting ability might boost B cell expansion and stimulate tumor-specific CD8^+^ T_RM_ cells. In EGFR-WT, the abundance of CD8^+^ T_RM_-like cells and their GZMB production were higher than those in EGFR-MT (Fig. 4h, i, k, m). Consequently, the increased proportion of CD8^+^ T_RM_-like cells can effectively lyse cancer cells by producing cytolytic materials such as GZMB.

To test our hypothesis, we first defined the area of TLS-like lesion (see “Methods” section) and then evaluated the abundance of T_FH_-like (CXCL13^+^CD4^+^), B (CXCR5^+^CD20^+^), and T_RM_-like (CD103^+^CD8^+^) cells within the TLS-like lesions of archival tumor tissue samples using multiplexed IF. Interestingly, we found that the total area of TLS-like lesion was significantly smaller in EGFR-MT than EGFR-WT (Fig. 6b, left and right upper panels and Supplementary Data 4a). For the validation set of 19 independent tumor samples used for multiplexed IF, we also observed a smaller area of TLS-like lesion in EGFR-MT than EGFR-WT (Fig. 6b, right lower panel and Supplementary Data 4c). In overall TLS-like lesion, the much less number of CD4^+^ and CD8^+^ T cells were found in EGFR-MT than EGFR-WT (Fig. 6c, d and Supplementary Figs. S10, 11). Notably, the numbers of T_FH_-like (CXCL13^+^CD4^+^) and T_RM_-like (CD103^+^CD8^+^) cells in EGFR-MT were significantly lower than EGFR-WT (Fig. 6c, d and Supplementary Figs. S10, 11, Supplementary Data 4b). We observed consistent results from the validation set of 19 independent tumor samples (Fig. 6e and Supplementary Fig. S12, Supplementary Data 4d). These results altogether suggest that CXCL13–CXCR5-mediated interactions between T_FH_–B and T_RM_–B cells scaffold T_FH_–B–T_RM_ lymphocyte cooperation in TLS for antitumor immunity in lung cancer as summarized in Fig. 6f.

## Discussion

In the present study, we performed comparative single-cell transcriptome analysis between EGFR-WT and EGFR-MT lung tumors across subsets of CD45^+^ immune cells using scRNA-seq to elucidate the underlying mechanisms of the different response rates to anti-PD-1 immunotherapy. Recently, accumulating evidence has indicated that activating the oncogenic pathway in tumor cells could diminish the antitumor immune response by modulating immune cell differentiation and recruitment^65^. Our study reveals the significant depletion of tumor-infiltrating B cells, CD4^+^ T_FH_-like cells, and CD8^+^ T_RM_-like cells in EGFR-MT tumors. In addition, T_FH_-like, B, and T_RM_-like cells of EGFR-MT showed a lower expression of genes involved in TLS formation, antigen presentation, and cytotoxicity, respectively, than EGFR-WT. EGFR-WT and EGFR-MT patient groups showed bias towards males with smoking history and female non-smokers, respectively, in both discovery and validation cohorts. Previous studies also reported a higher prevalence of *EGFR*-mutant NSCLC in female non-smokers than male smokers^66,67^. To test whether gender or smoking status was a confounder of the observed depletion of T_FH_-like, B, and T_RM_-like cells in EGFR-MT tumors, we compared CIBERSORTx scores for the three cell types between different smoking status or genders in EGFR-WT and EGFR-MT groups of the NSCLC tumors of TCGA cohort, separately. In neither EGFR-WT nor EGFR-MT group, we could observe a significant decrease of the three cell types in non-smokers and females (Supplementary Fig. S14a), which indicates that neither smoking status nor gender was a confounder of the observed association between EGFR mutation status and TME phenotypes. In addition, we observed only a weak correlation of T_RM_-like cell abundance or TLS area with PD-L1 expression (Supplementary Fig. S14b), which suggests that PD-L1 was not a confounder of our observed association between EGFR mutation status and TME phenotypes. Altogether, these results suggest that a higher intrinsic resistance of *EGFR*-mutant tumors to anti-PD-1 immunotherapy is primarily due to the depletion of these three lymphocyte subsets, major functional components of TLS for antitumor immunity, and their dysfunctional modulation in tumors. Furthermore, we found the disappearance of T_FH_–B and T_RM_–B interactions mediated by the CXCL13–CXCR5 axis in EGFR-MT tumors, which may negatively affect the formation of TLS in the tumor. Thus, targeting genes involved in these intra- and inter-cellular networks may improve the anti-PD-1 response in EGFR-MT patients.

Previous studies have demonstrated a correlation between the intratumoral abundance of T_RM_ cells and patient prognosis in lung cancer^49,68,69^. In this study, we demonstrate the feasibility of predicting the response to anti-PD-1 immunotherapy based on the proportion of T_RM_-like cells. Quantification of a specific intratumoral cell subset would be a useful biomarker for predicting clinical outcomes. Using the recently developed CIBERSORTx for gene expression deconvolution with single-cell transcriptome data, we extracted expression signatures for each cell subset in the lung tumor and found that CD8^+^ T_RM_-like cells were highly predictive of the anti-PD-1 response in two independent cohorts of NSCLC. Notably, we tested the expression signature of T_RM_-like cells constructed from lung tumors to identify anti-PD-1 responders in melanoma cohorts but observed no prediction power. Given that T_RM_ cells permanently reside in the peripheral tissue, these results are attributable to the tissue-specific nature of T_RM_ cells by adaptation to different local microenvironment.

In the present study, we employed Pearson residual analysis with mosaic plot visualization to systematically compare the cell subset distribution between sample groups (EGFR-WT and EGFR-MT tumors). Using the chi-square significance test with Bonferroni adjustment for multiple hypothesis tests, we could retrieve subsets showing proportional changes that were consistent with the observed phenotype differences between sample groups. The same analytical framework can be applied to various comparative single-cell transcriptome analyses between sample groups with distinct genotypes and phenotypes, and the interpretation of results is highly intuitive. The Pearson residual method can be applied in a comparative analysis between more than two groups, if not too many. Therefore, we anticipate expanded the applicability of the Pearson residual framework to single-cell transcriptome analysis in the future.

There are several limitations to this study. First, although we identified three cell subsets associated with antitumor immunity in the TLS area and their functional regulation, whether they are regulated independently or hierarchically coordinated remains unknown. For example, if CXCL13 produced by T_FH_-like cells promotes the persistence of T_RM_-like cells by interacting with recruited B cells, the transcriptional regulator of CXCL13 expression in T cells could be the primary target to improve an anti-PD-1 response in EGFR-MT tumors. Second, this study lacks data for myeloid cells (e.g., dendritic cells and myeloid-derived suppressor cells) and stromal cells (e.g., cancer-associated fibroblasts), which also contribute to cancer–immune interactions. Although they are not directly involved in killing cancer cells, their activity enhances or suppresses major cytotoxic lymphocytes through cytokine-mediated cell-to-cell communication and the presentation of tumor antigens. Therefore, a comparison of subsets of myeloid and stromal cells would reveal additional cellular components for determining the response to anti-PD-1 immunotherapy. Third, our study cohort has a bias toward the adenocarcinoma subtype of NSCLC (it includes only a single patient with the squamous carcinoma subtype). Thus, some findings from the present study are possibly relevant to the adenocarcinoma subtype only. Fourth, two patients with G719A (P5) and G719S (P8) were included in the present study. Although G179X are considered sensitizing mutations, these were not included in a phase 3 trial comparing osimertinib with other EGFR-TKIs for EGFR-mutant NSCLC patients as first line (FLAURA)^3^ and are under-represented in clinical trials compared to exon 19del and L858R. Fifth, although we demonstrated that smoking status and genders are not confounders of the observed decrease of TLS in EGFR mutants, we may not rule out other confounding factors. Much large sample sets of genomically and clinically annotated tumors will provide a more comprehensive assessment of our findings. Finally, although we demonstrated the importance of homeostasis of T_RM_-like cells for antitumor immunity in TME of NSCLC through single-cell RNA velocity and co-regulatory network analysis, we could not provide functional validations in the present study. Thus, we need to conduct further experimental analyses in vitro and in vivo conditions to confirm our proposed mechanisms in future study.

In conclusion, we performed a comparative single-cell transcriptome analysis between *EGFR*-mutant and wild-type lung tumors and found several key lymphocyte subsets involved in the mechanisms underlying the anti-PD-1 response. In particular, we elucidated that T_FH_–B–T_RM_ lymphocyte cooperation is critical to antitumor immunity in lung cancer. Dysregulation of the homeostasis of T_RM_ cell state and T_FH_–B and T_RM_–B crosstalk involved in TLS development in EGFR-MT lung tumor may account for unfavorable responses to the antitumor immunotherapy with PD-1 blockades. Our findings provide guidelines for developing strategies to enhance anti-PD-1 efficacy in patients with *EGFR* mutations.

## Methods

### Tumor specimen collection and preparation

Patients diagnosed with NSCLC and who underwent surgical resection at Severance Hospital (Seoul, Republic of Korea), between June 2018 and August 2018, were prospectively enrolled. We consecutively enrolled five patients with EGFR-MT and five patients with EGFR-WT lung cancer. As depicted in Supplementary Data 1, six patients were in pathological stage I, two in stage II, and two in stage III; all patients with EGFR-MT were female never smokers, and those with EGFR-WT were males with a smoking history. This bias regarding the contribution of gender and smoking history to *EGFR* mutation has been previously reported in Asian patients with lung cancer^66,67^. No patient had undergone chemotherapy or radiation therapy before surgical resection. The study was approved by the Institutional Review Board of Severance Hospital (IRB No. 4-2018-1210, 4-2014-0775, and 4-2020-0945). We have obtained written informed consents from all patients in this study.

We collected fresh tumor tissues on the day of the surgery, which were mechanically and enzymatically dissociated using a gentleMACS dissociator (Miltenyi Biotec, Gladbach Bergisch, Germany, Cat# 130-093-235) and the Human Tumor Dissociation Kit (Miltenyi Biotec, Cat# 130-095-929) following the manufacturer’s instructions. After incubation for 1 h at 37 °C, the resuspended samples were filtered through a 70-µm-MACS SmartStrainer (Miltenyi Biotec, Cat# 130-098-462) into RPMI-1640 medium (Corning, Inc., Corning, NY, USA) supplemented with 10% fetal bovine serum (Biowest, Riverside, MO, USA) and centrifuged at 300 × *g* for 10 min. The pellet was suspended in PBS containing 2% fetal bovine serum. For fluorescence-activated cell sorting (FACS) analysis, single-cell suspensions were stained for 20 min at 4 °C with the following fluorescent-dye-conjugated antibodies: LIVE/DEAD™ Fixable Red Dead Cell Stain Kit (Invitrogen, Carlsbad, CA, USA, Cat# L23102. 1:100) and CD45 (Biolegend, San Diego, CA, USA, clone 2D1. 1:100). The samples were sorted using a FACS Aria III sorter (BD Biosciences, Franklin Lakes, NJ, USA) into live and single CD45^+^ cells. The sorted cell population was analyzed to ensure successful cell sorting, as shown in Supplementary Fig. S1. Cell viability was assessed by the Luna FL Fluorescence Cell Counter (Logos Biosystems, Anyang, Korea, Cat#L20001) and confirmed to be 75–98% in all samples.

### 10× single-cell RNA sequencing

Single-cell samples for constructing libraries and sequencing were prepared using a Chromium controller according to the 10× Single Cell 3′ v2 protocol (10× Genomics, Pleasanton, CA, USA). To acquire 10,000 targeted cells, the cells were diluted in nuclease-free water and mixed with the master mix. The single-cell suspensions were loaded with Single Cell 3′ Gel Beads and Partitioning Oil (GEM) into a Single Cell 3′ Chip. To generate the GEMs, single cells were uniquely barcoded, and cDNAs were used to construct libraries according to the manufacturer’s instructions. The libraries were sequenced using a HiSeq 2500 sequencing system (Illumina, San Diego, CA, USA).

### Single-cell RNA sequencing data analysis

From the raw FASTQ files of scRNA-seq data (forward/reverse strands and barcode sequence), we used Cell Ranger analysis pipelines (v2.1.1) to obtain a gene-count matrix. We used the Seurat version3.0^17,70^ pipeline to analyze scRNA-seq data. For quality control, we filtered cells with a higher percentage of mitochondrial genes or those showing outlier behavior in nfeatures_RNA and nCount_RNA from the “FeatureScatter” plot for each sample as recommended. We then normalized the data using the “NormalizeData” function with the recommended setting (log-normalization scale factor of 10,000). The normalized samples were integrated with 7431 highly variable genes by integration functions (“FindIntegrationAnchors” and “IntegrationData”) with default settings. For dimension reduction and visualization of the integrated data, “ScaleData,” “RunPCA,” and “RunUMAP” functions were used with default settings. Unsupervised clustering was performed by “FindNeighbors,” which conducts shared-nearest-neighbor-based clustering with 2000 highly variable genes and “FindClusters,” which conducts Louvain clustering (dimension of 20 and resolution of 0.65). The parameters for UMAP plotting before batch correction were the same as those for the integrated one.

### Comparative single-cell transcriptome analysis based on Pearson residual

To investigate proportional changes between sample groups (e.g., EFGR-WT and EGFR-MT) across cell subsets defined by clustering, we evaluated deviations in the observed cell count for each group from the expected count for a subset using Pearson residual:

$${{{{{{\mathrm{Pearson}}}}}}}\,{{{{{{\mathrm{residual}}}}}}}\,({r}_{{ij}})=\frac{{O}_{{ij}}\,-{E}_{{ij}}\,}{\sqrt{{E}_{{ij}}\,}},$$

where *i* and *j* represent indices for each group and cell subsets, respectively, and *O* and *E* represent the observed and expected cell counts, respectively. The expected cell count (*E*) for a group *i* of a subset *j* was calculated by the following equation:

$${E}_{{ij}}=\frac{{T}_{i}}{{T}_{{{{{{{\mathrm{tot}}}}}}}}}\cdot {T}_{j},$$

where *T*_tot_, *T*_*i*_*, T*_*j*_ represent total cell count for the entire data set, total cell count for a group *i*, and total cell count for a subset *j*, respectively. The advantage of the Pearson residual is that the sign of the residual indicates the direction of the difference of the observed count from the expected count (i.e., positive for augmentation and negative for depletion compared to expected count). Pearson residual (*r*) follows an approximately normal distribution; thus, scores larger than 2 or smaller than −2 are significant by *p* < 0.05. For a highly conservative statistical test along with Bonferroni correction for multiple hypothesis test adjustment, we used a significance threshold of *p*-value = 0.00056 (*p*-value of 0.01 was divided by the number of subsets, 18, for Bonferroni correction). Thus, we considered only cell subsets with *r* > 3.5 (augmentation) and *r* < −3.5 (depletion) for follow-up functional interpretation. We visualized the results of the goodness of fit test using a mosaic plot, in which subsets with deviation in observed cell counts from the expected cell counts are indicated by blue (augmentation), red (depletion), or gray (no significant change) colors. The goodness of fit for all subsets was also evaluated by the chi-square statistic (*p*-value).

### Categorizing cell subsets into major immune cell types

We used marker gene expression to categorize cell clusters into immune cell types. The following list of marker genes was adapted from the Reference Gene Expression Profiles (RGEP)^71^: *CD3D*, *CD3E*, *CD3G*, and *CD28* for T cells; *CD4* for CD4^+^ T cells; *CD8A* and *CD8B* for CD8^+^ T cells; *FOXP3* and *IL2RA* for Treg cells; *FCGR3A*, *FCGR3B*, *NCAM1*, *KLRB1*, *KLRC1*, *KLRD1*, *KLRF1*, and *KLRK1* for NK and NKT cells; *CD14*, *CD68*, *CD163*, and *C8F1R* for macrophages and monocytes; *CD19*, *MS4A1*, *CD79A*, *CD79B*, and *BLNK* for B cells; *IL3RA*, *CLEC4C*, and *NRP1* for dendritic cells; and *KIT* and *IL1RL1* for ILC2 cells. The expression of these marker genes was visualized as violin plots (Supplementary Fig. S2) and validated on UMAP (Supplementary Figs. S3–9).

### Differentially expressed gene analysis with single-cell transcriptome data

We performed two types of DEG analysis using the “FindMarkers” function. The “group-DEG” was evaluated by comparing the EGFR-WT group to the EGFR-MT group for each subset. The “subset-DEG” was evaluated by comparing one subset to all other subsets within a major cell type (i.e., NK and NKT cells, CD4^+^ T cells, CD8^+^ T cells). DEGs were accepted if their respective *q*-value was smaller than 0.05 and |avgLogFC| > 0.25 (default threshold by Seurat package). We excluded genes with high expression but a low ratio of non-zero cells.

### Multiplex immunofluorescence staining

We cut 4-μm-thick tissue sections from formalin-fixed paraffin-embedded blocks, and then performed multiplexed IF for the nine patients analyzed by scRNA-seq, except P3 that was not available owing to the insufficient quantities. Tissue slides were heated for at least 1 h in a dry oven at 60 °C, dewaxed using xylene, and evaluated by multiplex immunofluorescence staining with a Leica Bond Rx™ Automated Stainer (Leica Biosystems, Wetzlar, Germany). Briefly, the slides were heated for 30 min and dewaxed with Leica Bond Dewax solution (#AR9222), followed by antigen retrieval with Bond Epitope Retrieval 2 (#AR9640, Leica Biosystems) in pH 9.0 solution for 30 min. Each primary antibody was incubated for 30 min, followed by detection using the polymer HRP Ms+Rb secondary antibody (ARH1001EA, Akoya Biosciences, Marlborough, MA, USA) for 10 min. To evaluate the CD103, GZMB in CD8^+^ T cells, primary antibodies against the following antigens were used: CD8 (MCA1817, Bio-Rad, Hercules, CA, USA, 1:300), CD103 (ab129202, Abcam, Cambridge, UK, 1:500), and GZMB (262A-15, Cell Marque, Rocklin, CA, USA, 1:50). Visualization of each antibody was accomplished by treatment with Opal 520 TSA Plus, Opal 620 TSA Plus, and Opal 780 TSA Plus, respectively, for 10 min, following which the slide was treated with Bond Epitope Retrieval 1 (#AR9961, Leica Biosystems) for 20 min to remove bound antibodies. To evaluate the CXCL13^+^CD4, CXCR5^+^CD20, CD103^+^CD8, we performed another set of multiplexed IF using primary antibodies against the following antigens: for CD4 (ab133616, Abcam, Cambridge, UK, 1:200), CD8 (MCA1817, Bio-Rad, Hercules, CA, USA, 1:300), CXCL13 (PA5-47035, Invitrogen, Massachusetts, USA, 1:40), CXCR5 (72172 S, Cell Signaling Technology, Massachusetts, USA, 1:200), CD20 (ab9475, Abcam, Cambridge, UK, 1:100), and CD103 (ab129202, Abcam, Cambridge, UK, 1:500). Visualization of each antibody was accomplished by treatment with Opal 570 TSA, Opal 520 TSA, Opal 690 TSA, Opal 620 TSA, Opal 480 TSA, Opal 780 TSA. Nuclei were visualized with DAPI, and the section was coverslipped using HIGHDEF^®^ IHC fluoromount (ADI-950-260-0025, Enzo, Farmingdale, NY, USA). To validate the results from the prior tumor samples, we conducted additional multiplex IF for tumor samples from 19 independent NSCLC patients (EGFR-WT, *n* = 9; EGFR-MT, *n* = 10) using CD8 (MCA1817, Bio-Rad, Hercules, CA, USA, 1:300), CD103 (ab129202, Abcam, Cambridge, UK, 1:500), CXCL13 (PA5-47035, Invitrogen, Massachusetts, USA, 1:40), CD4 (ab133616, Abcam, Cambridge, UK, 1:200), CD20 (ab9475, Abcam, Cambridge, UK, 1:100) and CK (NBP2-29429, NOVUS, Centennial, USA, 1:200). Visualization of each antibody was accomplished by treatment with Opal 570 TSA, Opal 520 TSA, Opal 690 TSA, Opal 620 TSA, Opal 480 TSA, and Opal 780 TSA.

### Image acquisition and quantitative data analysis

Slides were scanned using a Vectra Polaris Automated Quantitative Pathology Imaging System (Akoya Biosciences), and images were analyzed using InForm 2.2 software and TIBCO Spotfire™ (Akoya Biosciences). To acquire reliable unmixed images, representative slides of each emission spectrum and unstained tissue slide were used. Each stained section was used to establish the spectral library of fluorophores required for multispectral analysis as a reference for target quantitation, as the intensity of each fluorescent target was extracted from the multispectral data by linear unmixing. Each cell was identified by detecting nuclear spectral elements (DAPI). The immune cell population from each panel was characterized and quantified using the cell segmentation tool in InForm image analysis software. For co-expression analysis, the data obtained from InForm were sent to Spotfire™ software, and the threshold for positivity of each factor was determined using IF scoring methods. All cells on each slide were designated as positive or negative for each antibody, and the data were categorized and exported to a Microsoft Excel spreadsheet file for analysis. We used the Spotfire™ program, after tissue and cell segmentation, and compared the expression intensity, and assessed our results based on the cutoff values. The number of positive cells was counted on each slide. Immune subsets including GZMB^+^ CD103^+^CD8^+^, CD103^+^CD8^+^, CD8^+^, CXCL13^+^CD4^+^, and CD4^+^ T cells were quantified by cell counts per mm^2^ in both the tumor nest and stroma based on the whole slide. Pathologists reviewed each slide stained with multiplex IF and H&E of nine patients and manually marked TLS-like lesion based on the colocalization of CD20^+^ B cells, CD4^+^, CD8^+^ T cells. For the total area of the TLS-like lesions in both the tumor nest and stroma of each sample, we also evaluated the counts of CD103^+^CD8^+^, CD8^+^, CXCL13^+^CD4^+^, CD4^+^ T cells, and CD20^+^ B cells.

### Flow cytometry analysis

Flow cytometry was performed using CytoFLEX (Beckman Coulter, Brea, CA, USA). Data were analyzed using FlowJo software (Tree Star, Ashland, OR, USA). For immunolabeling TILs, fluorophore-conjugated monoclonal antibodies against the following proteins were used: CD8 (RPA-T8, Cat# 301048, Biolegend, San Diego, CA, USA, 1:50), CD3 (SK7, Cat# 344808, Biolegend, San Diego, CA, USA, 1:100), PD-1 (EH12.2H7, Cat# 329933, Biolegend, San Diego, CA, USA, 1:20), CD103 (Ber-ACT8, Cat# 350230, Biolegend, San Diego, CA, USA, 1:20), CD39 (A1, Cat# 328210, Biolegend, San Diego, CA, USA, 1:20), GZMB (QA16A02, Cat# 372214, Biolegend, San Diego, CA, USA, 1:50), and CD4 (RPA-T4, Cat# 560837, BD Biosciences, San Diego, CA, USA, 1:50). The LIVE/DEAD Fixable Near-IR Dead Cell Stain Kit was from Invitrogen (L34973, Waltham, Massachusetts, USA, 1:100). The sequential gating strategies used for analysis for data obtained from flow cytometry are described in Supplementary Fig. S15.

For *t*-distributed stochastic neighbor embedding (tSNE) visualization of flow cytometry data, we used three samples for each group (EGFR-WT and EGFR-MT). Each sample was down-sampled to 7000 randomly selected CD3^+^ live and singlet-gated cells, yielding 42,000 cells. A tSNE plot for the merged 42,000 cells was constructed using FlowJo (v10.6.2) with default settings (3000 iterations and perplexity = 100).

### RNA velocity-based estimation of cellular transition probability

We performed RNA velocity analysis by velocyto^52^ using the default pipeline. We processed single-cell transcriptome data for EGFR-WT and EGFR-MT tumors separately. We used the pre-built UMAP coordinate of CD8^+^ T cells from the Seurat package as the embedding coordinates for plotting. The transition probability was obtained from the “transition_prob.T” variable. RNA velocity predicts the future mRNA abundance of each gene from the ratio of spliced and unspliced mRNA levels based on a steady-state model. For ScVelo^53^ (ver 0.2.3) analysis which enables estimation of RNA velocity without steady-state assumption, we used the default pipeline. Loom files for splicing and un-splicing count information for each cell were obtained from RNA velocity analysis. The transition probability was obtained from the “scv.utials.get_transition_matrix()” class. The future state of each cell is predicted based on all genes. For a given subset of cells, the probability of transition to another subset can be calculated for each cell. The mean values of transition probability for each subset of cells were obtained by averaging the corresponding cell transition to other cell subsets.

### MetaCell-based analysis of gene expression correlations

The correlation coefficient calculated from single-cell gene expression data was markedly low, mainly because of the zero-inflation property of scRNA-seq data. The sparsity of single-cell expression profiles can be compensated by imputation approaches, but they generate many false gene-gene correlations^72^. For more sensitive and robust detection of co-expression between two genes, we employed MetaCell^57^, which pools *k* nearest neighbor cells. We collected the raw count matrix from the Seurat object of single-cell data for EGFR-WT and EGFR-MT groups, separately. Each data set was processed using default parameter settings in the MetaCell pipeline without a cell filtering step: T_vm = 0.08 for mcell_gset_filter_varmean; T_tot = 100 and T_top3 = 2 for mcell_gset_filter_cov; K = 100 and dsamp = T for mcell_add_cgraph_from_mat_bknn; min_mc_size = 20, p_resamp = 0.75, and n_resamp = 500 for mcell_coclust_from_graph_resamp; K = 30, min_mc_size = 30, and alpha = 2 for mcell_mc_from_coclust_balanced; and T_lfc = 3 for mcell_mc_split_filt. The pooled cell “metacell” was generated with a corrected expression matrix calculated by the regularized geometric mean of each “metacell.” We calculated Spearman’s rank correlation coefficient between two genes based on the corrected expression matrix.

### Determining the abundance of T_RM_-like cells in tumor samples using expression signature

Expression deconvolution technology was used to estimate the proportion of the T_RM_-like cells from bulk RNA-seq profiles for tumor samples. To construct a gene expression profile of T_RM_-like cells, we employed CIBERSORTx^58^, a recently developed tool for expression deconvolution that can utilize single-cell transcriptome data. We first generated a signature matrix of the 7431 highly variable genes based on the scRNA-seq data. We then deconvoluted bulk RNA-seq data using default parameter settings for each tumor sample obtained from LUAD and lung squamous cell carcinoma (LUSC) of TCGA cohort data set^73^ to estimate the proportion of T_RM_-like cells. We also estimated the proportion of T_RM_-like cells of tumor samples from two other public cohorts of patients with lung cancer treated with anti-PD-1 immunotherapy (GSE126045 and GSE135222)^60,61^.

### Analysis of receptor–ligand-mediated cell–cell interactions

We used CellPhoneDB^63^ to analyze the receptor–ligand-mediated cell–cell interactions. We used the raw count matrix with the 7431 highly variable genes based on the scRNA-seq data for cells of C0–14 clusters as input data. We ran the EGFR-WT and EGFR-MT data separately with the default pipeline and parameters.

### Reporting summary

Further information on research design is available in the Nature Research Reporting Summary linked to this article.

## Supplementary information

Supplementary Information

Description of Additional Supplementary Files

Supplementary Data 1

Supplementary Data 2

Supplementary Data 3

Supplementary Data 4

Supplementary Data 5

Supplementary Data 6

Supplementary Data 7

Supplementary Data 8

Reporting Summary

## Data Availability

The single-cell RNA sequencing data generated in this study have been deposited in the Gene Expression Omnibus database (GSE144945). TCGA cohort data set were obtained from [https://gdac.broadinstitute.org]^59^. Bulk RNA-seq data for two public cohorts of patients with lung cancer treated with anti-PD-1 immunotherapy were obtained from GSE126045^60^ and GSE135222^61^. The remaining data are available within the Article, Supplementary Information, or Source Data file. Source data are provided with this paper.

## Source data

Source Data
