# Supplementary material for: Dysregulation of TFH-B-TRM lymphocyte cooperation is associated with unfavorable anti-PD-1 responses in EGFR-mutant lung cancer
Source: Nat Commun. 2021 Oct 18;12:6068. doi: 10.1038/s41467-021-26362-0 (PMC8523541; doi:10.1038/s41467-021-26362-0)
Supplement: Supplementary file 1 — Supplementary Information [file 41467_2021_26362_MOESM1_ESM.pdf]

Supplementary Figures

Dysregulation of T_{FH}-B-T_{RM} lymphocyte cooperation is associated with unfavorable anti-PD-1 responses in *EGFR*-mutant lung cancer

Jae-Won Cho[1], Seyeon Park[2], Gamin Kim[3], Heonjong Han[1], Hyo Sup Shim[4], Sunhye Shin[3], Yong-Soo Bae[5], Seong Yong Park[6*], Sang-Jun Ha[2*], Insuk Lee[1*], Hye Ryun Kim[3*]

[1]Department of Biotechnology, College of Life Science and Biotechnology, Yonsei University, Seoul 03722, Korea

[2]Department of Biochemistry, College of Life Science and Biotechnology, Yonsei University, Seoul 03722, Korea

[3]Division of Medical Oncology, Department of Internal Medicine, Yonsei Cancer Center, Yonsei University College of Medicine, Seoul 03722, Korea

[4]Department of Pathology, Yonsei University College of Medicine, Seoul 03722, Korea

[5]Department of Biological Sciences, Science Research Center (SRC) for Immune Research on Non-lymphoid Organ (CIRNO), Sungkyunkwan University, Jangan-gu, Suwon, Gyeonggi-do, 16419, South Korea

[6]Department of Thoracic and Cardiovascular Surgery, Yonsei University College of Medicine, Seoul 03722, Korea

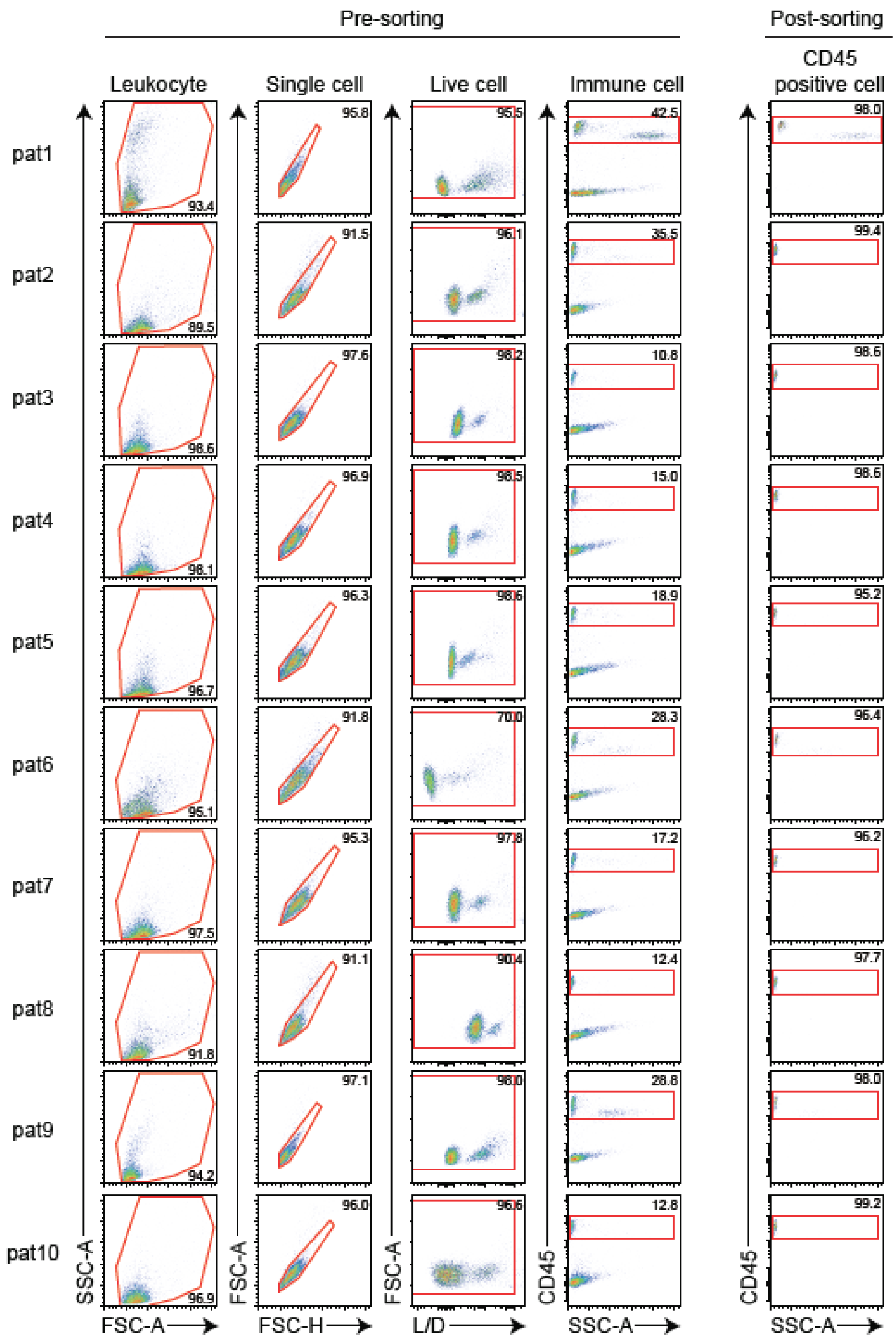

Figure S1. Sorting of tumor-infiltrating leukocytes from tumor tissues of individual NSCLC patients. Graphs depicting the pre-sorting plots (left) and post-sorting plots (right) of each patient's tumor tissue. Leukocytes are identified by forward scatter-area (FSC-A) versus side scatter-area (SSC-A) plot. Gating the plot by forward scatter-area (FSC-A) against forward scatter-height (FSC-H) indicates single cells. Live cells were separated by forward side scatter-area (FSC-A) and LIVE/DEAD™ Stain Kit. Immune cells are sorted by gating on CD45-positive. The purity of the sorted cells was greater than 97%.

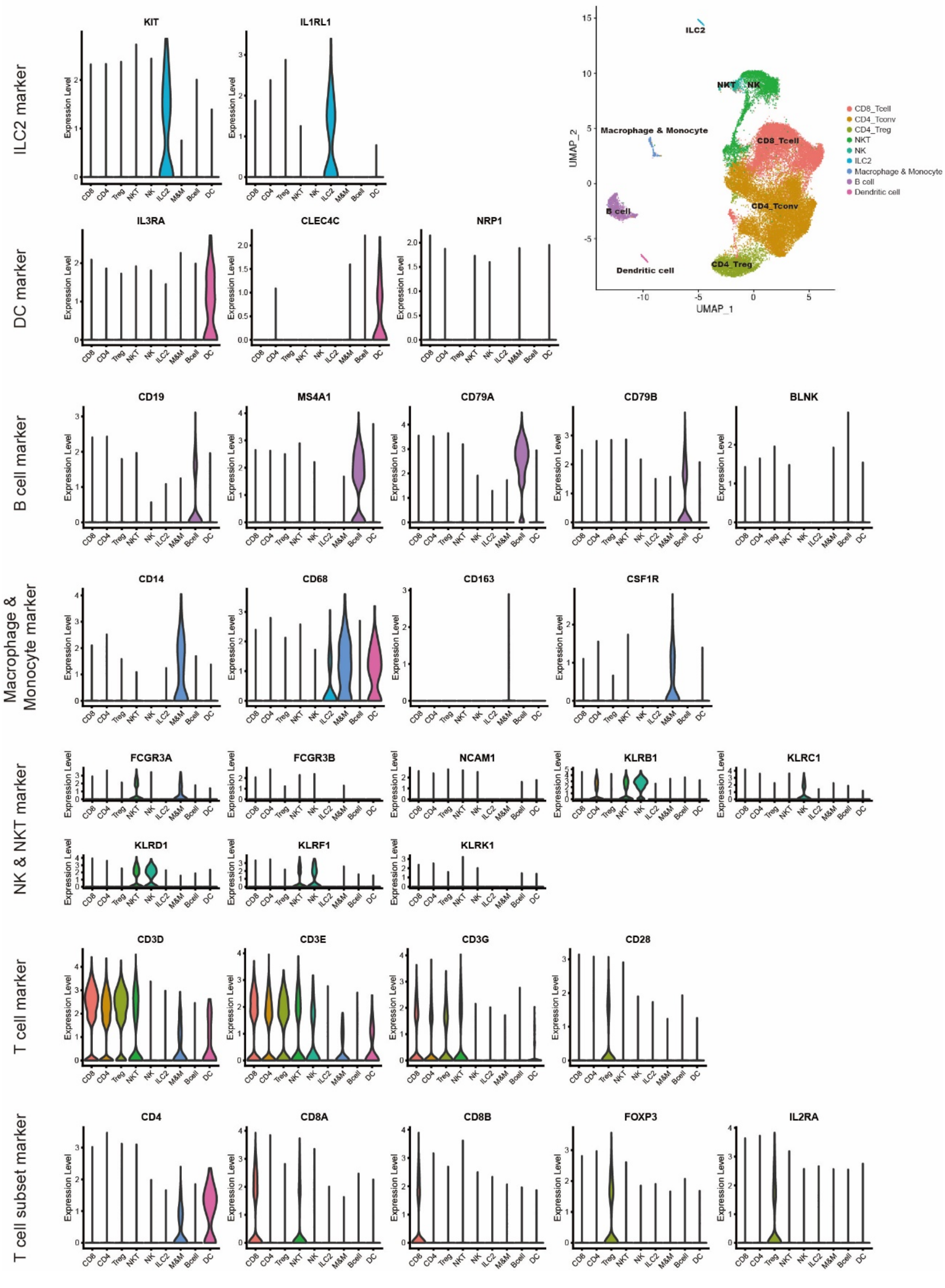

Figure S2. Marker gene expression analyses. Annotation of each cluster based on the expression of canonical marker genes for major immune cell types.

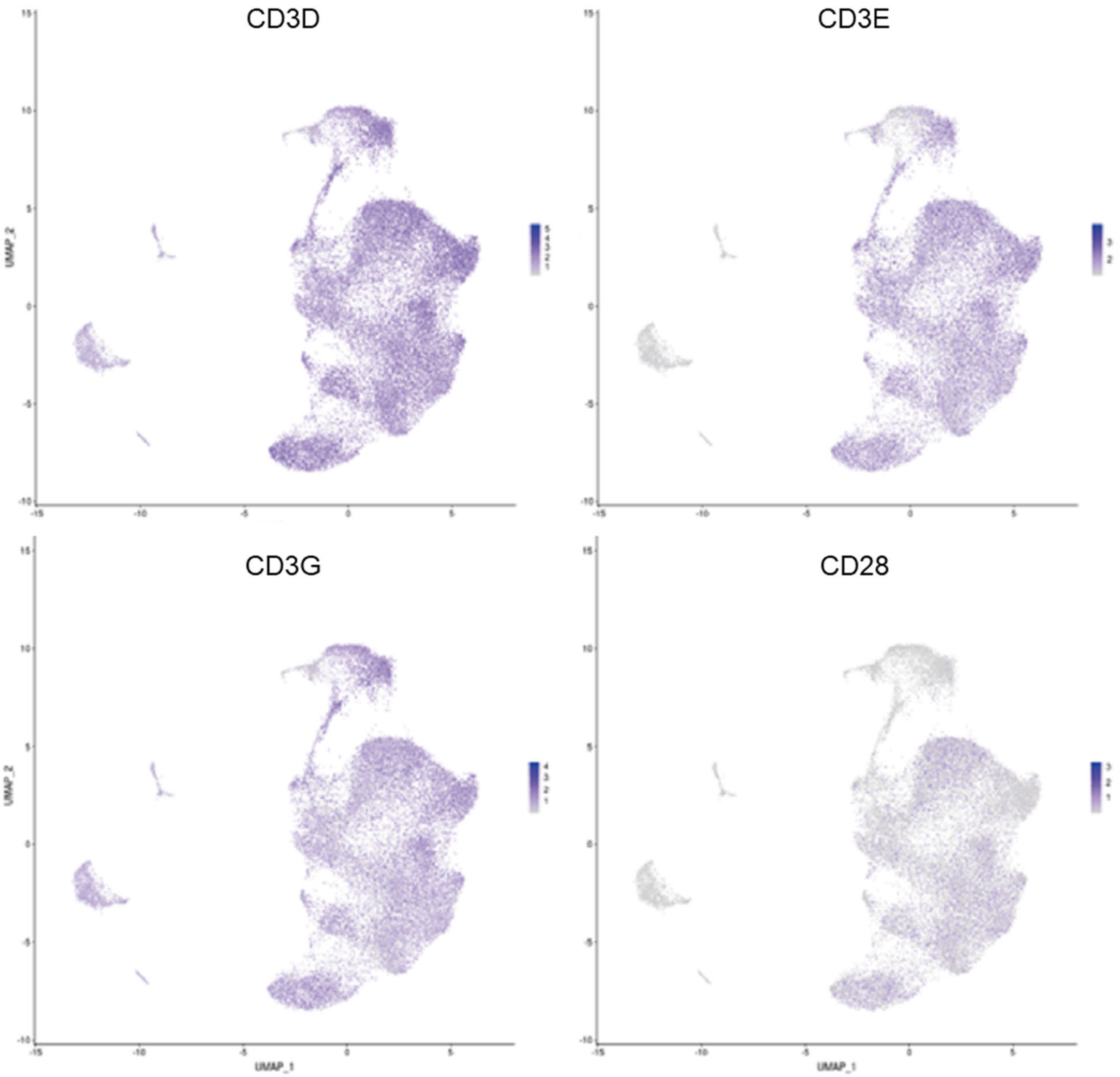

Figure S3. Marker gene expressions for T cells. We tested CD3D, CD3E, CD3G, and CD28 to identify T cells. Significant overexpression was observed in CD3D (q < 2.22E-308), CD3E (q = 4.65E-204), and CD28 (q = 1.65E-153) from CD4 T cell clusters and CD3D (q < 2.22E-308), CD3E (q = 3.84E-264), and CD3G (q = 3.31E-123) in CD8 T cell clusters, respectively (DEG analysis result: two-sided, Bonferroni correction).

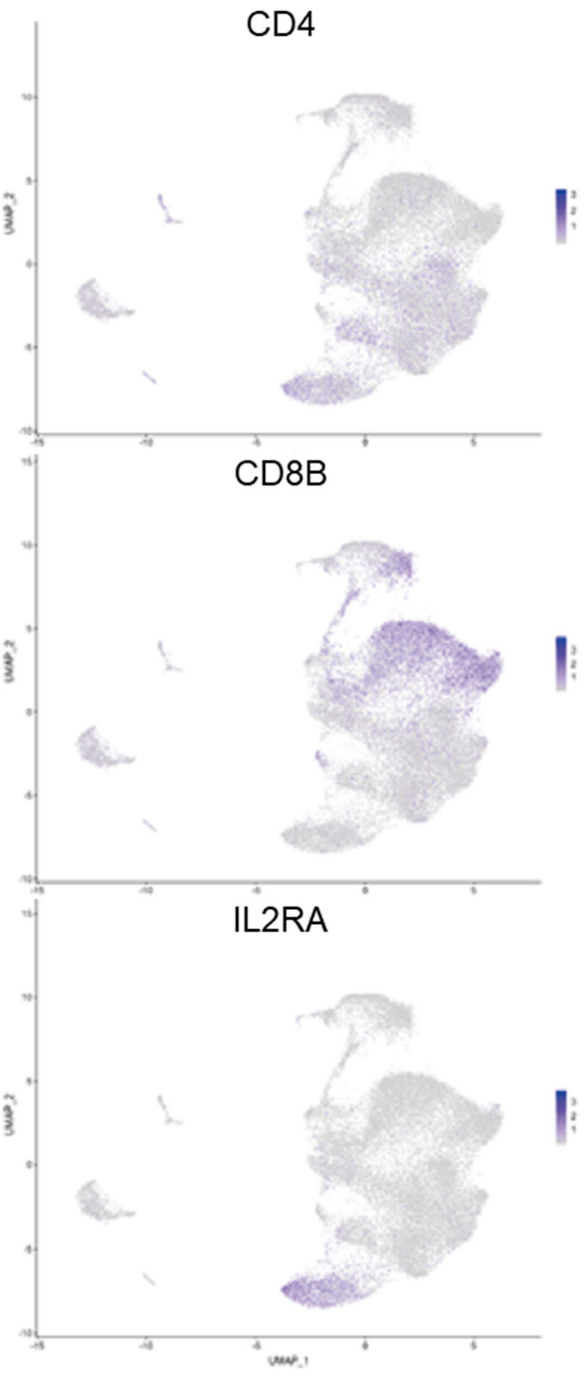

Figure S4. Marker gene expressions for T cell subtypes. We tested CD4, CD8A, CD8B, FOXP3, and IL2RA to identify T cell subtypes. For CD4 T cells, the expression level of CD4 T cells was slightly higher than that of other immune cells (*p*-value: ~3E-5, avglogfc: ~0.19). However, because it has been reported that the RNA expression level of CD4 is low in scRNA-seq data, we defined those clusters as CD4 T cells[1]. For CD8 T cells, CD8A ($q < 2.22E-308$) and CD8B ($q = 7.69E-260$) were significantly overexpressed. CD4 Treg, CD4 ($q = 1.35E-273$), FOXP3 ($q < 2.22E-308$), and IL2RA ($q < 2.22E-308$) were significantly overexpressed (DEG analysis result: two-sided, Bonferroni correction).

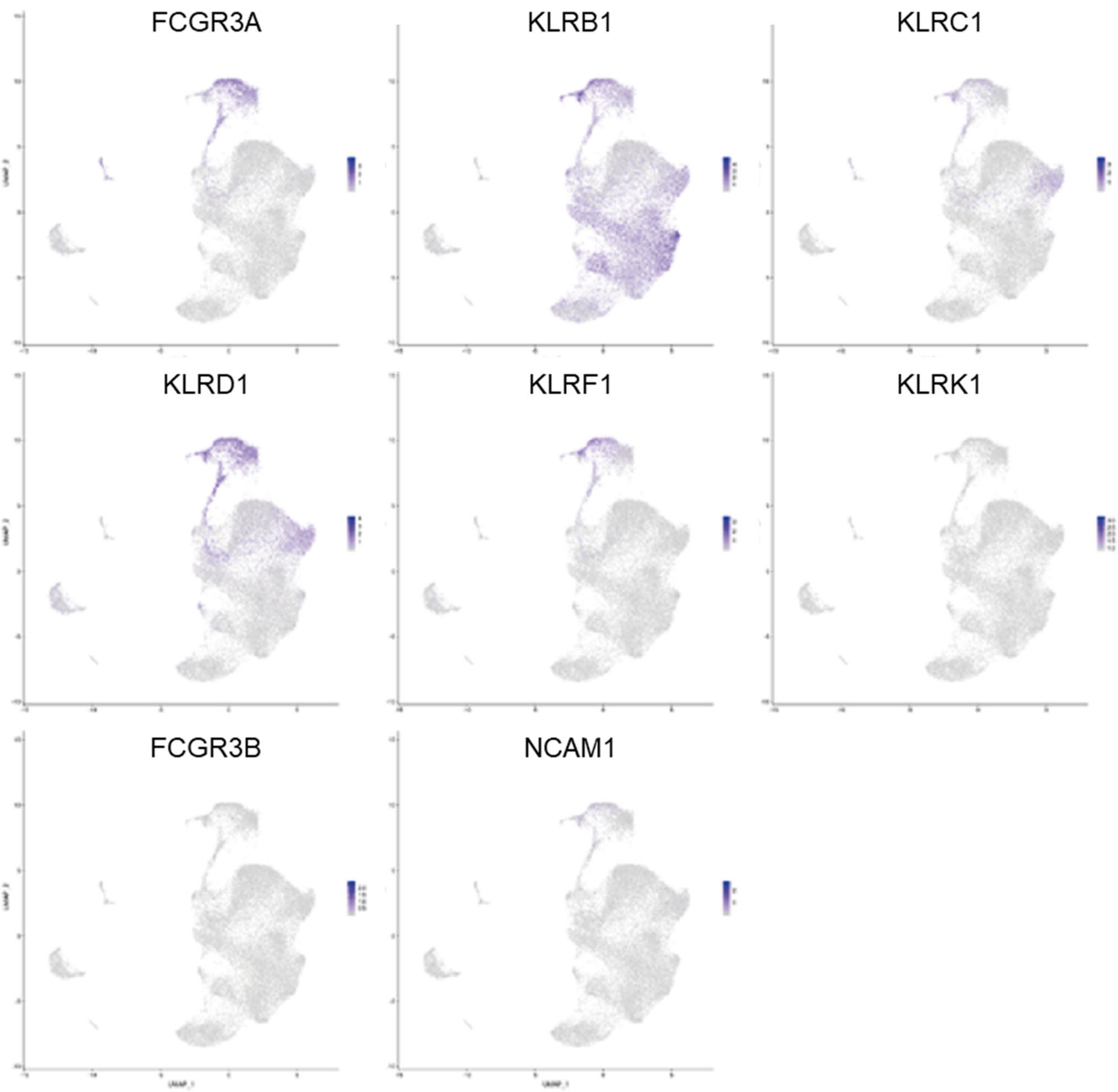

Figure S5. Marker gene expressions for NK or NKT cells. We tested FCGR3A, KLRB1, KLRC1, KLRD1, KLRF1, KLRK1, FCGR3B, and NCAM1 to identify NK or NKT cells. We observed significant overexpression in FCGR3A (q < 2.22E-308), KLRB1 (q = 9.90E-147), KLRD1 (q < 2.22E-308), and KLRF1 (q < 2.22E-308) from NK cells and FCGR3A (q < 2.22E-308), KLRB1 (q = 9.90E-147), KLRD1 (q < 2.22E-308), and KLRF1 (q < 2.22E-308) from NKT cells, respectively In addition, CD3D (q = 2.37E-206), CD3E (q = 2.89E-165), and CD3G (q = 2.04E-134) were overexpressed in NKT cells (Fig. S3). (DEG analysis result: two-sided, Bonferroni correction).

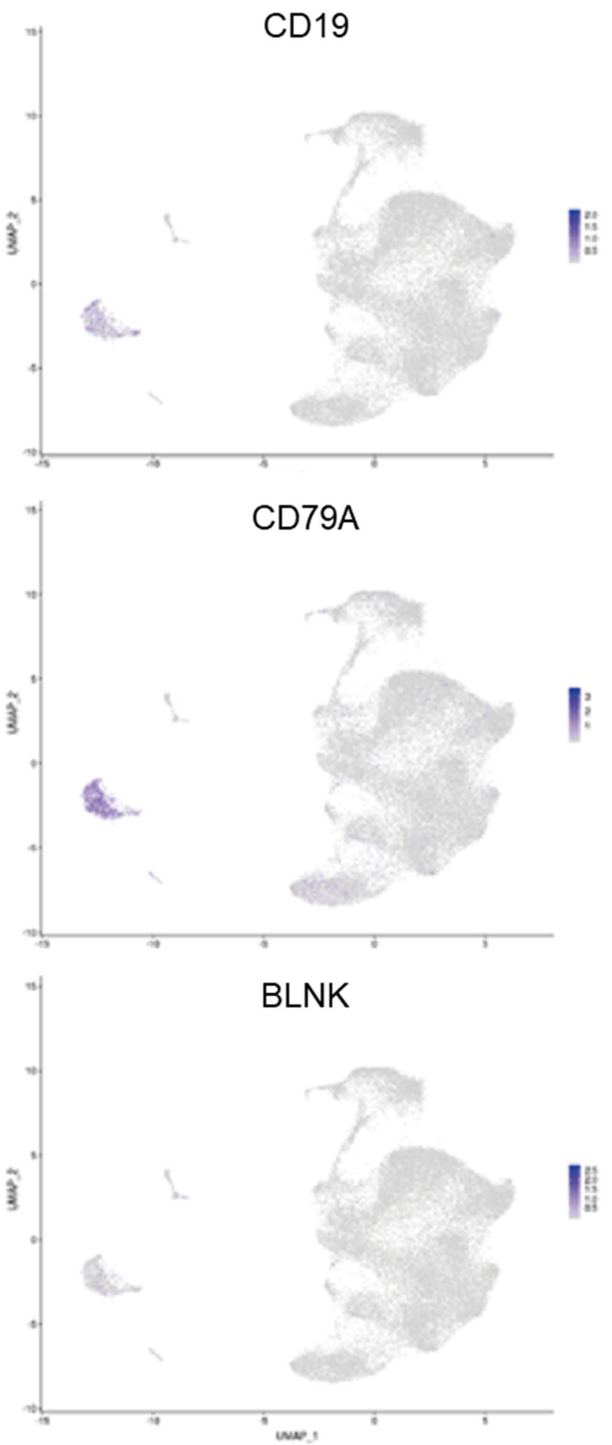

Figure S6. Marker gene expression in B cells. We tested CD19, MS4A1, CD79A, CD79B, and BLNK to identify B cells. We observed significant overexpression in CD19, MS4A1 (q < 2.22E-308), CD79A (q < 2.22E-308), and CD79B (q < 2.22E-308) (DEG analysis result: two-sided, Bonferroni correction).

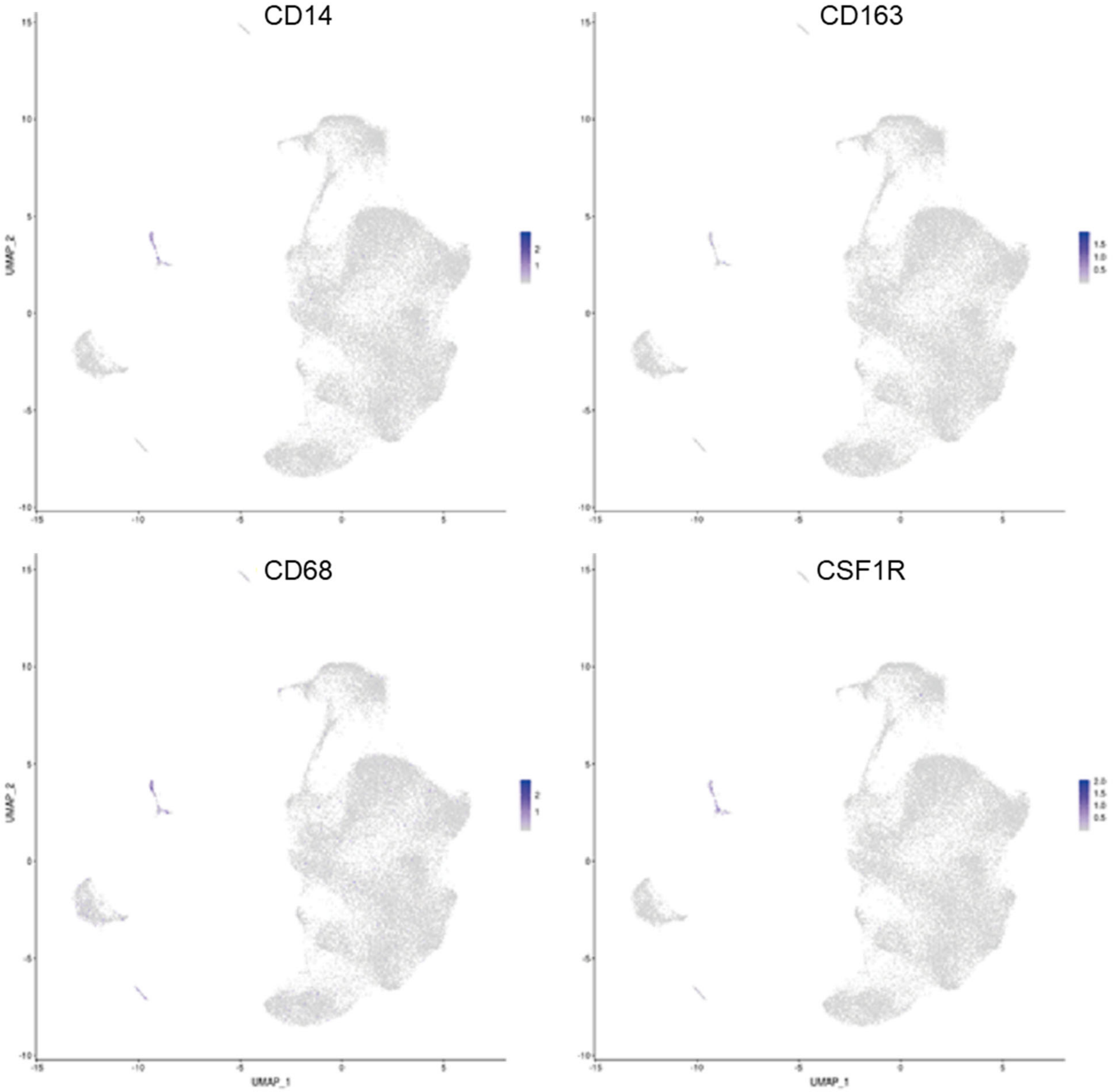

Figure S7. Marker gene expressions for macrophages and monocytes. We tested CD14, CD163, CSF1R, and CD68 to identify macrophages and monocytes. We observed significant overexpression in CD14 ($q < 2.22E-308$), CD68 ($q < 2.22E-308$), and CSF1R ($q < 2.22E-308$) (DEG analysis result: two-sided, Bonferroni correction). Unfortunately, we could not discriminate between macrophages and monocytes because of the similarity between these two cell types.

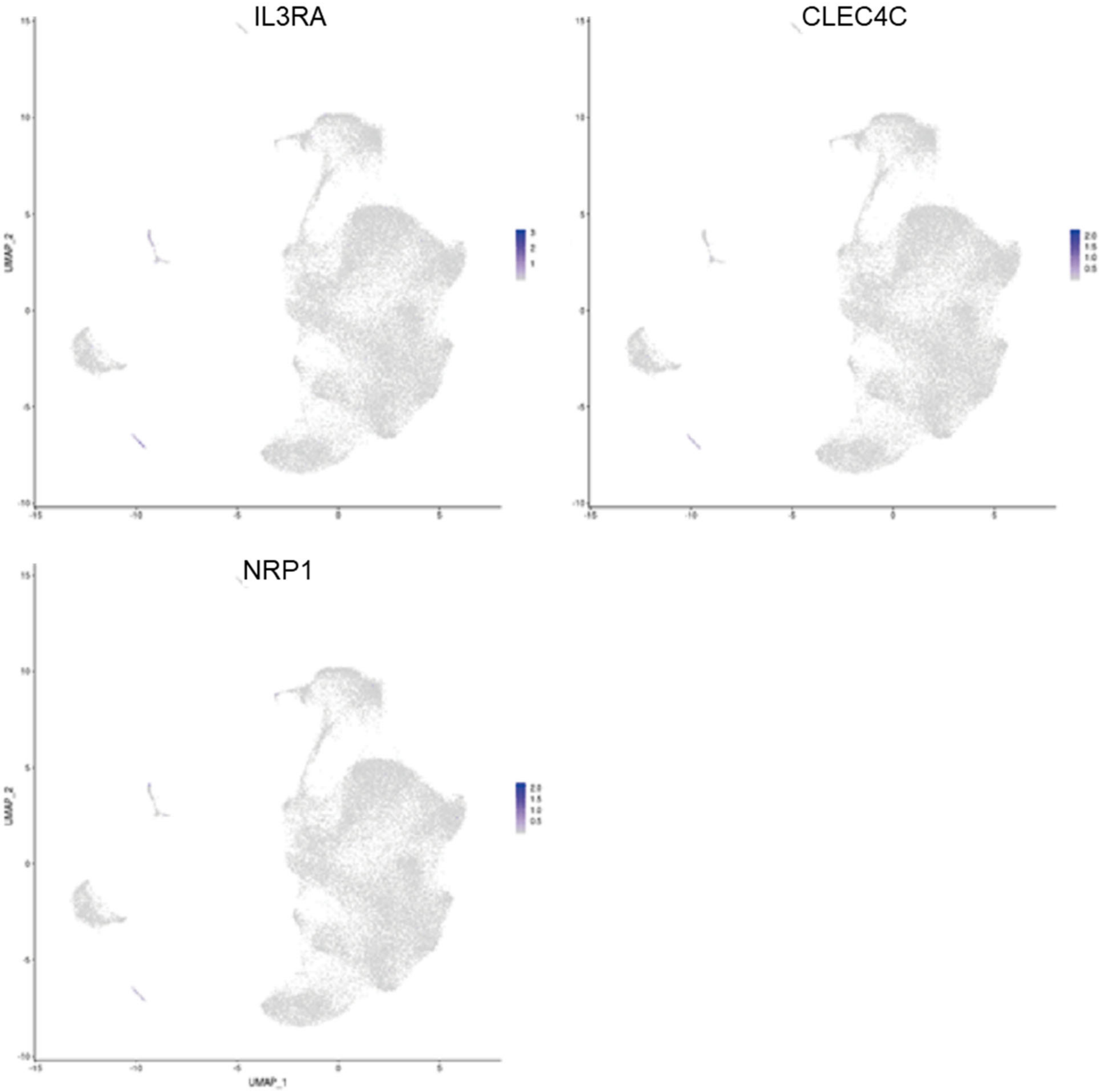

Figure S8. Marker gene expression for dendritic cells. We tested IL3RA, CLEC4C, and NRP1 to identify dendritic cells. We observed significant overexpression in IL3RA (q < 2.22E-308) and CLEC4C (q < 2.22E-308) (DEG analysis result: two-sided, Bonferroni correction).

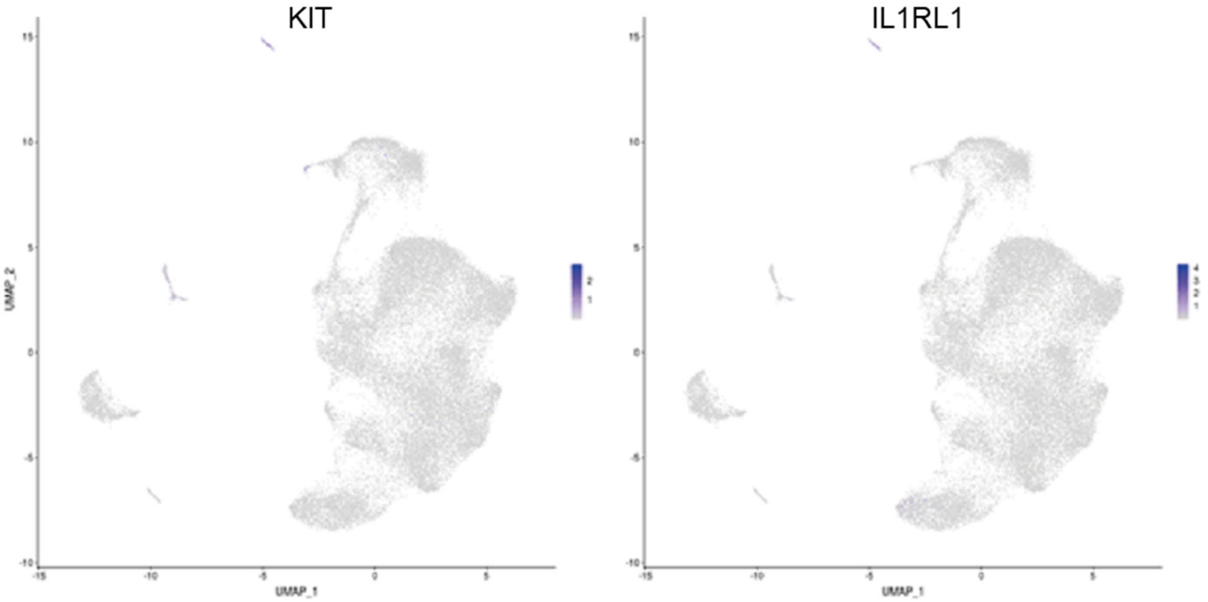

Figure S9. Marker gene expression for innate lymphoid cells (ILC2). We tested KIT and IL1RL1 (ST2) to identify ILC2. We observed significant overexpression in KIT ($q < 2.22E-308$) and IL1RL1 ($q < 2.22E-308$) (DEG analysis result: two-sided, Bonferroni correction).

EGFR-MT (P2)

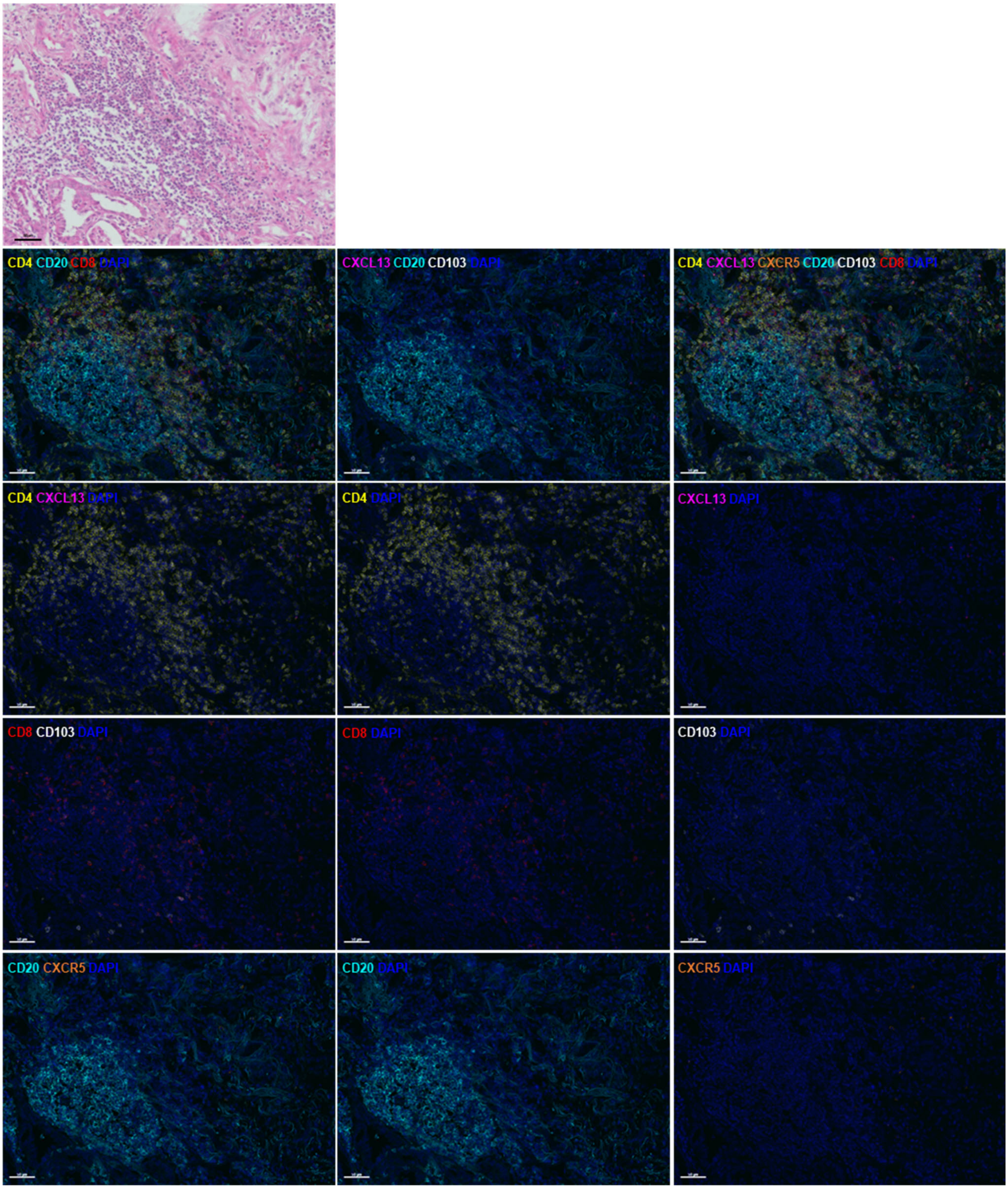
CD4 CD20 CD8 DAPI
CXCL13 CD20 CD103 DAPI
CD4 CXCL13 CXCR5 CD20 CD103 CD8 DAPI
CD4 CXCL13 DAPI
CD4 DAPI
CXCL13 DAPI
CD8 CD103 DAPI
CD8 DAPI
CD103 DAPI
CD20 CXCR5 DAPI
CD20 DAPI
CXCR5 DAPI

EGFR-MT (P5)

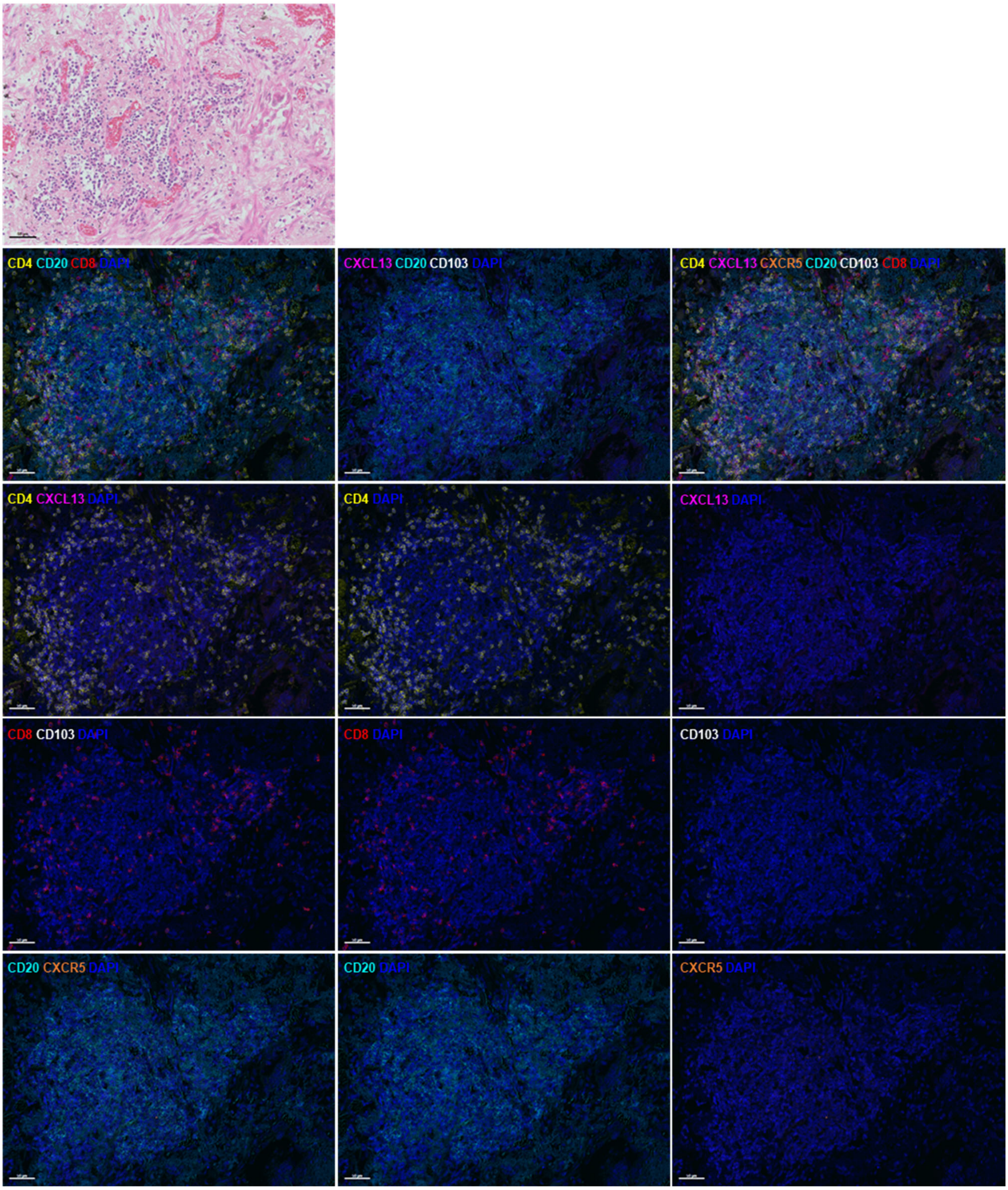
CD4 CD20 CD8 DAPI
CXCL13 CD20 CD103 DAPI
CD4 CXCL13 CXCR5 CD20 CD103 CD8 DAPI
CD4 CXCL13 DAPI
CD4 DAPI
CXCL13 DAPI
CD8 CD103 DAPI
CD8 DAPI
CD103 DAPI
CD20 CXCR5 DAPI
CD20 DAPI
CXCR5 DAPI

EGFR-MT (P7)

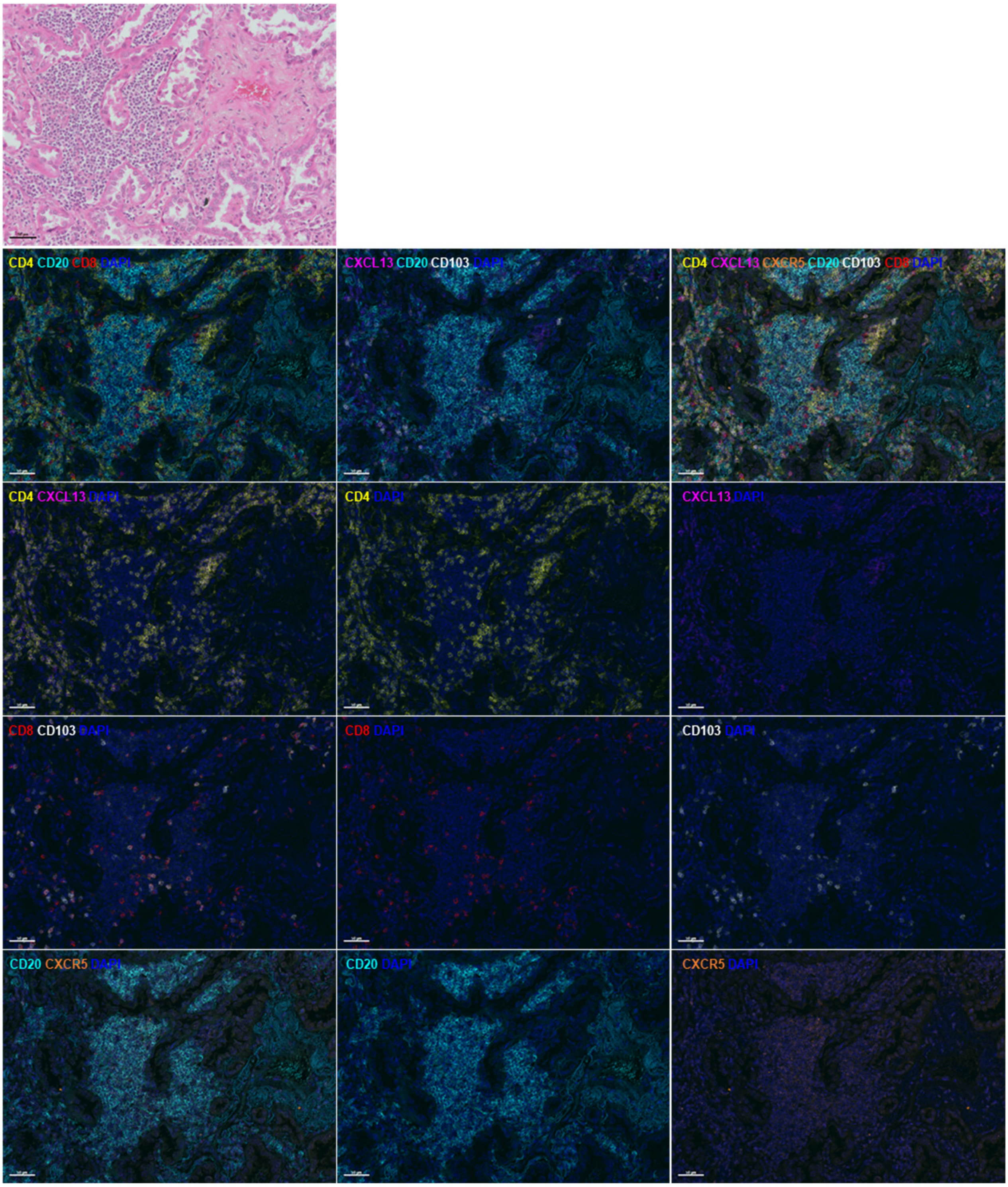

CD4 CD20 CD8 DAPI
CXCL13 CD20 CD103 DAPI
CD4 CXCL13 CXCR5 CD20 CD103 CD8 DAPI
CD4 CXCL13 DAPI
CD4 DAPI
CXCL13 DAPI
CD8 CD103 DAPI
CD8 DAPI
CD103 DAPI
CD20 CXCR5 DAPI
CD20 DAPI
CXCR5 DAPI

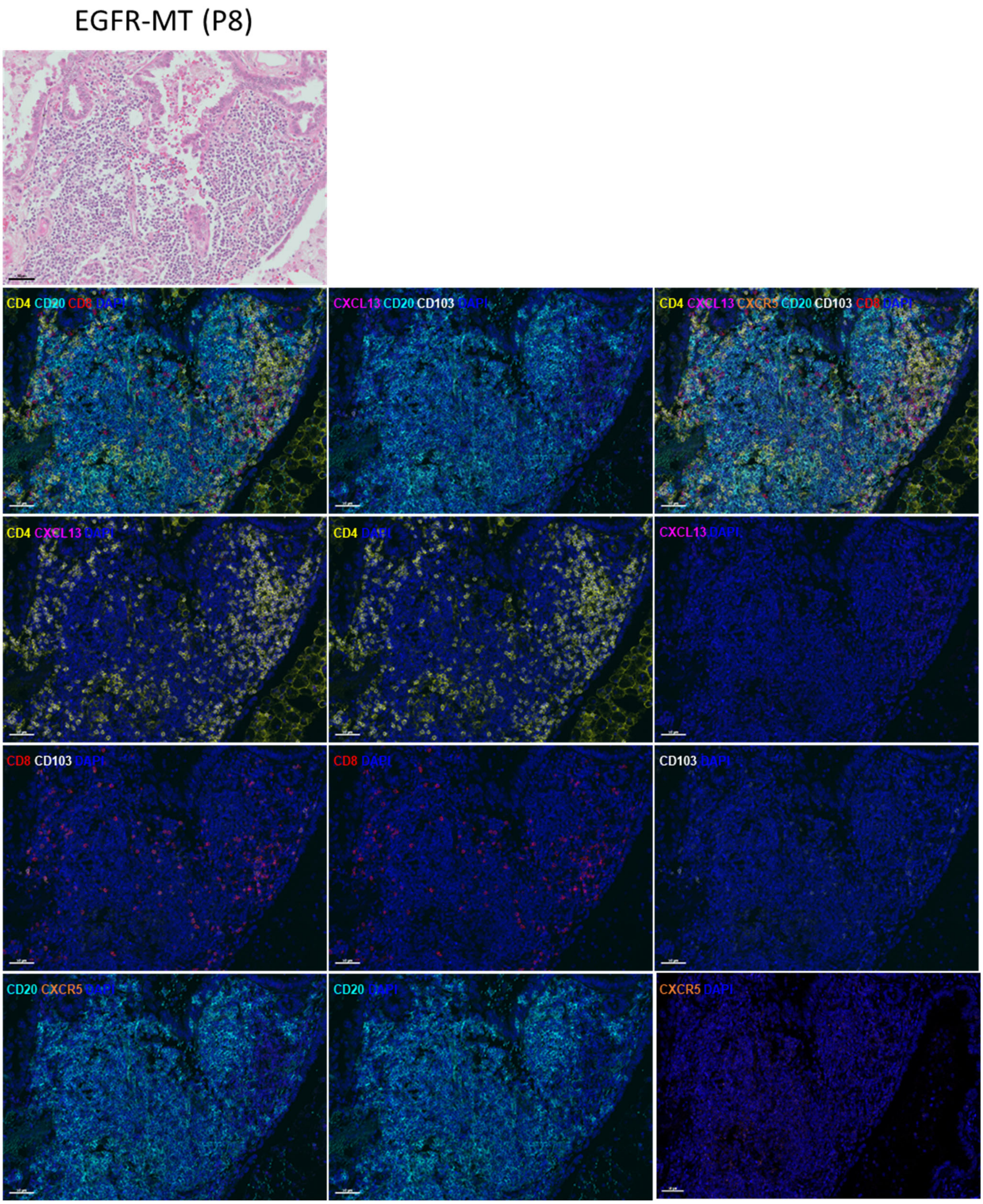

Figure S10. Representative multiplexed IF (scale bar 50μm) of CD4, CXCL13, CD20, CD103, CD8 in tumor specimens from EGFR-MT histology image with H&E staining (n=4).

EGFR-WT (P1)

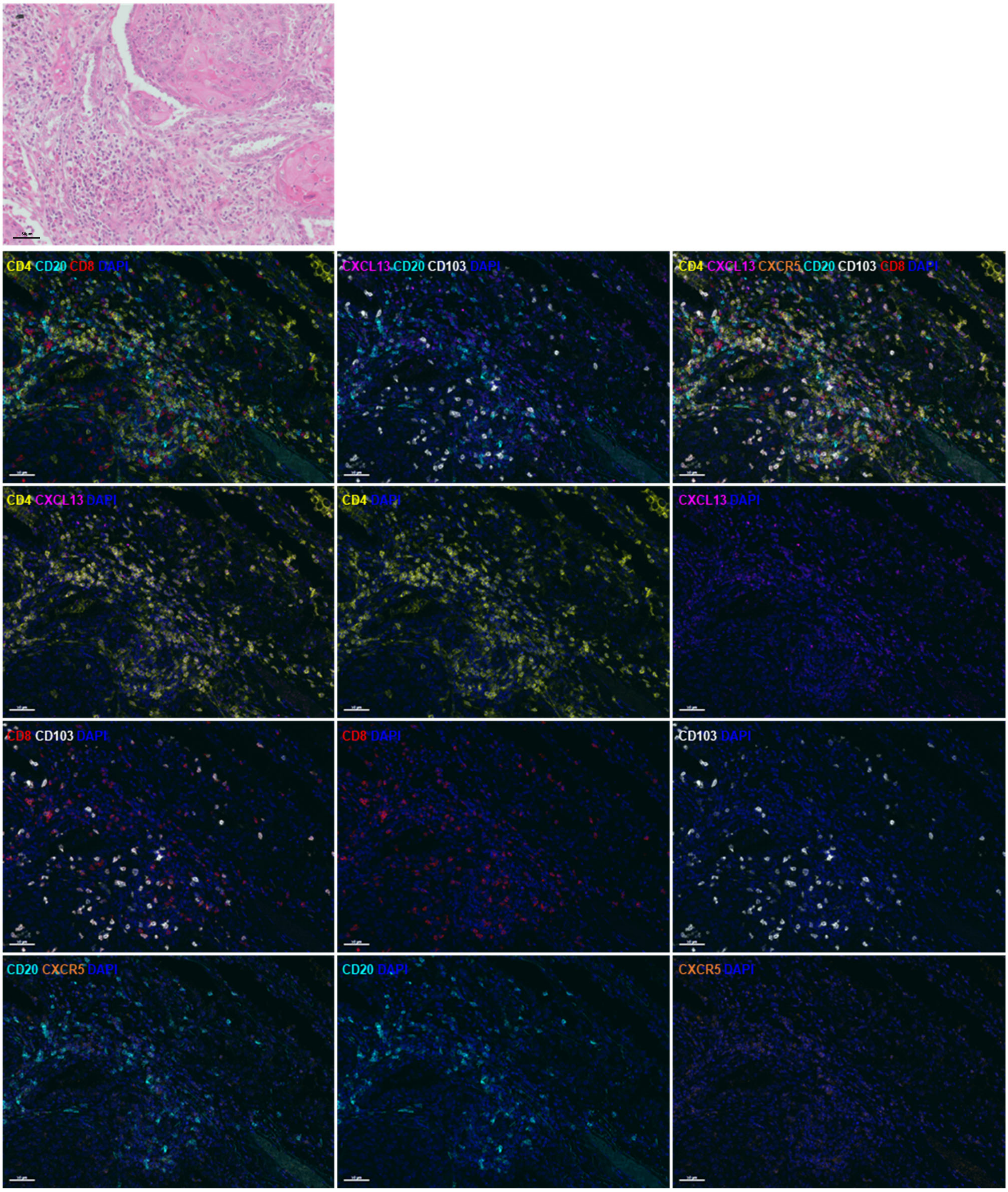
CD4 CD20 CD8 DAPI
CXCL13 CD20 CD103 DAPI
CD4 CXCL13 CXCR5 CD20 CD103 CD8 DAPI
CD4 CXCL13 DAPI
CD4 DAPI
CXCL13 DAPI
CD8 CD103 DAPI
CD8 DAPI
CD103 DAPI
CD20 CXCR5 DAPI
CD20 DAPI
CXCR5 DAPI

EGFR-WT (P4)

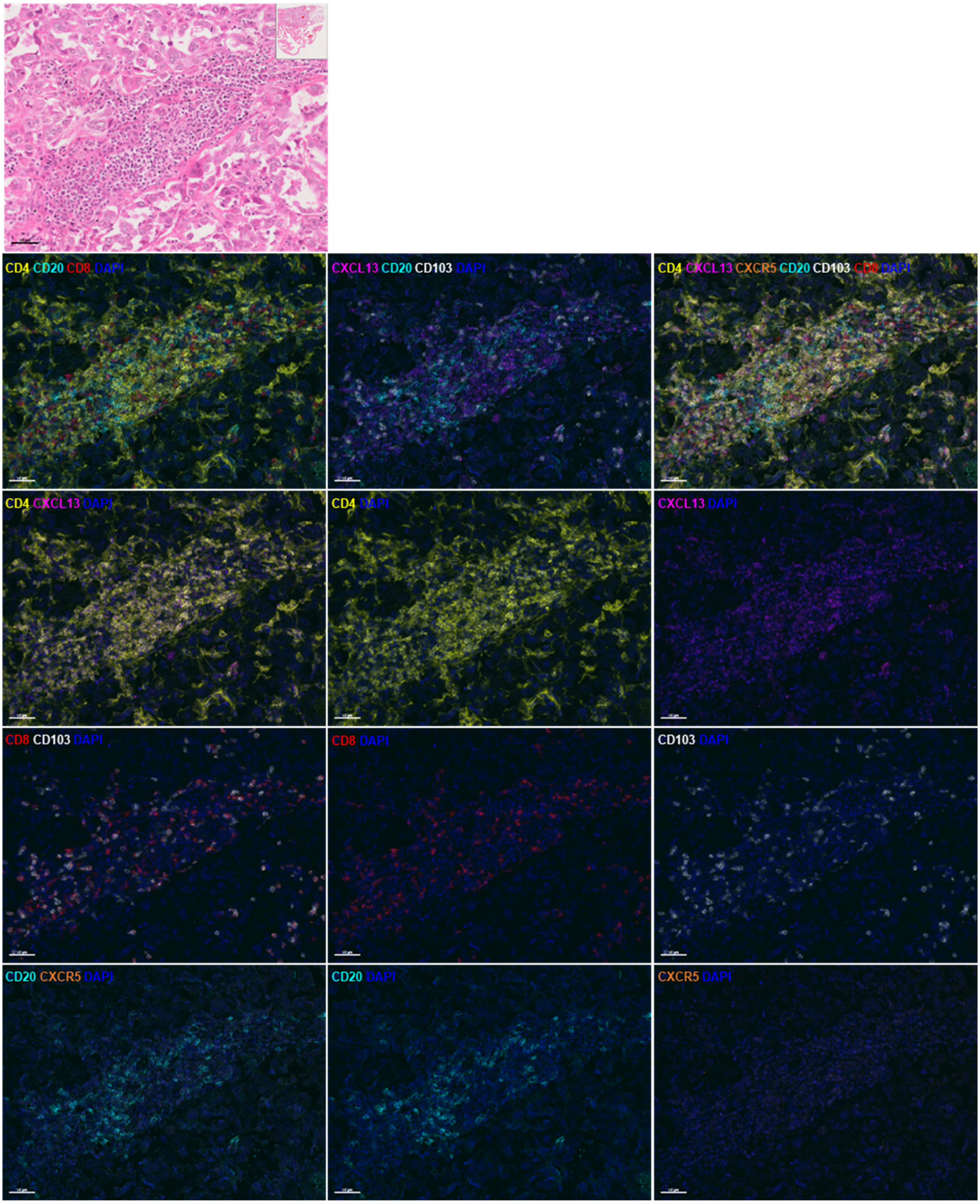
CD4 CD20 CD8 DAPI
CXCL13 CD20 CD103 DAPI
CD4 CXCL13 CXCR5 CD20 CD103 CD8 DAPI
CD4 CXCL13 DAPI
CD4 DAPI
CXCL13 DAPI
CD8 CD103 DAPI
CD8 DAPI
CD103 DAPI
CD20 CXCR5 DAPI
CD20 DAPI
CXCR5 DAPI

EGFR-WT (P6)

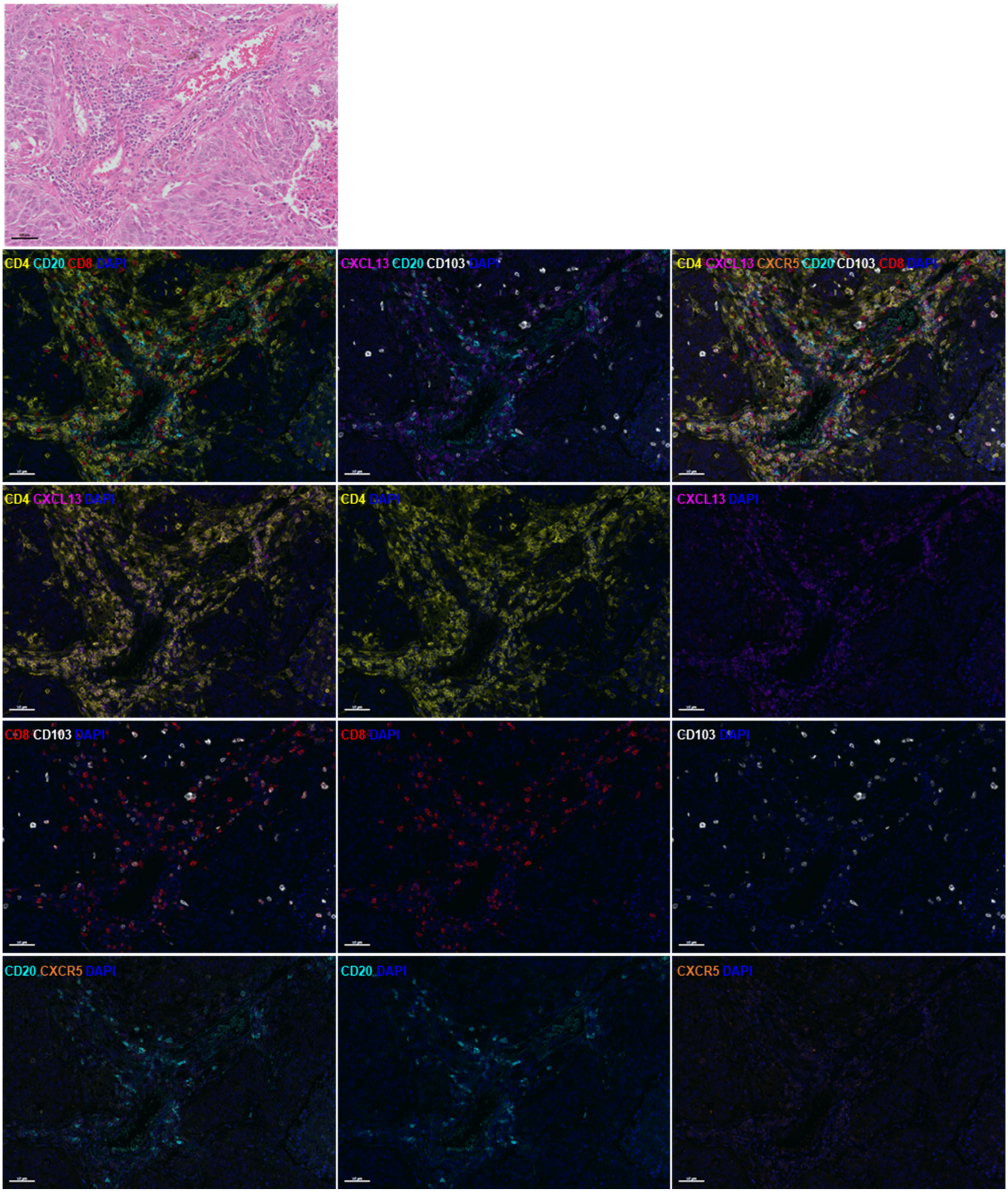
CD4 CD20 CD8 DAPI
CXCL13 CD20 CD103 DAPI
CD4 CXCL13 CXCR5 CD20 CD103 CD8 DAPI
CD4 CXCL13 DAPI
CD4 DAPI
CXCL13 DAPI
CD8 CD103 DAPI
CD8 DAPI
CD103 DAPI
CD20 CXCR5 DAPI
CD20 DAPI
CXCR5 DAPI

EGFR-WT (P9)

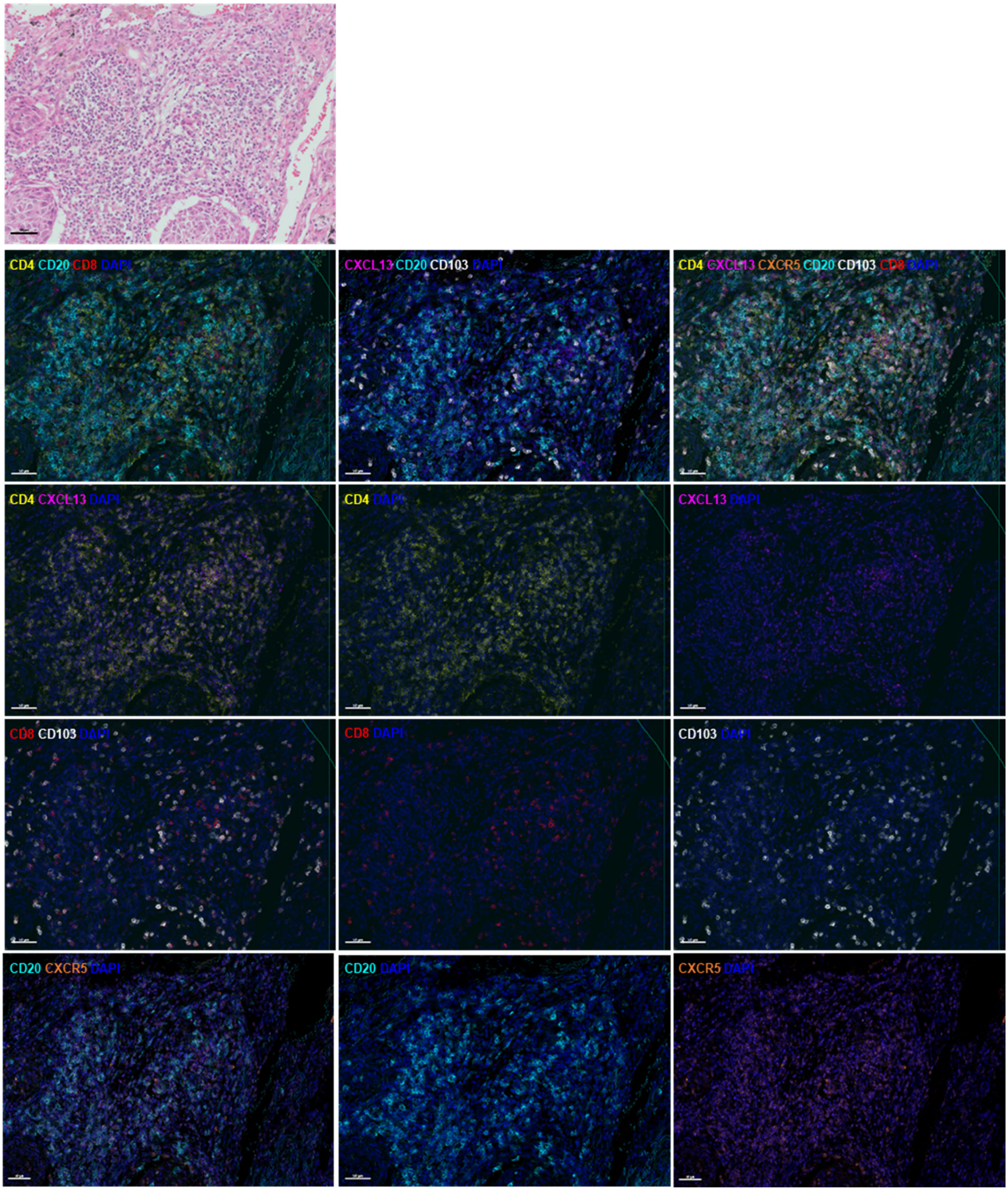
CD4 CD20 CD8 DAPI
CXCL13 CD20 CD103 DAPI
CD4 CXCL13 CXCR5 CD20 CD103 CD8 DAPI
CD4 CXCL13 DAPI
CD4 DAPI
CXCL13 DAPI
CD8 CD103 DAPI
CD8 DAPI
CD103 DAPI
CD20 CXCR5 DAPI
CD20 DAPI
CXCR5 DAPI

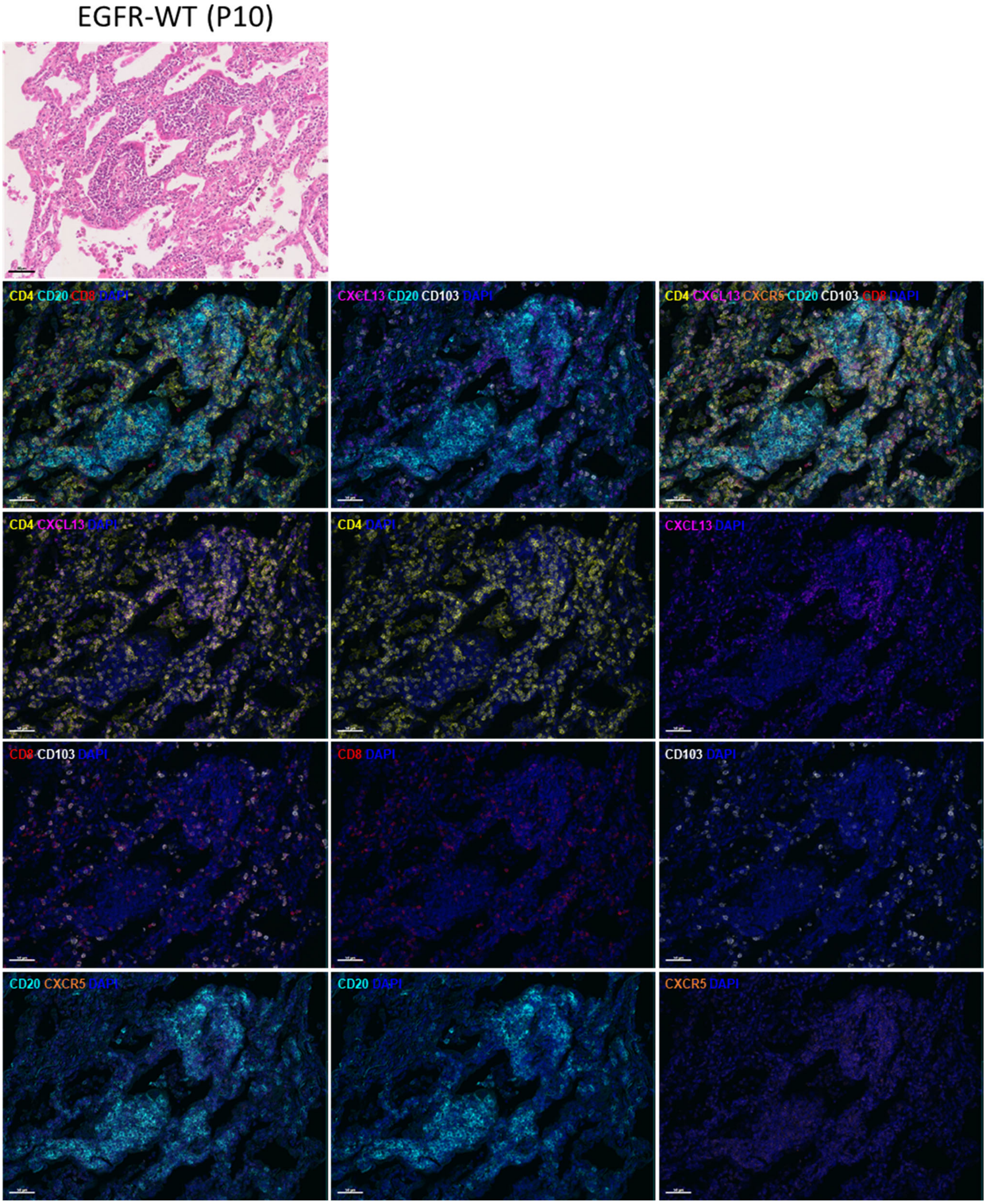

Figure S11. Representative multiplexed IF (scale bar 50µm) of CD4, CXCL13, CD20, CD103, CD8 in tumor specimens from EGFR-WT and histology image with H&E staining (n=5).

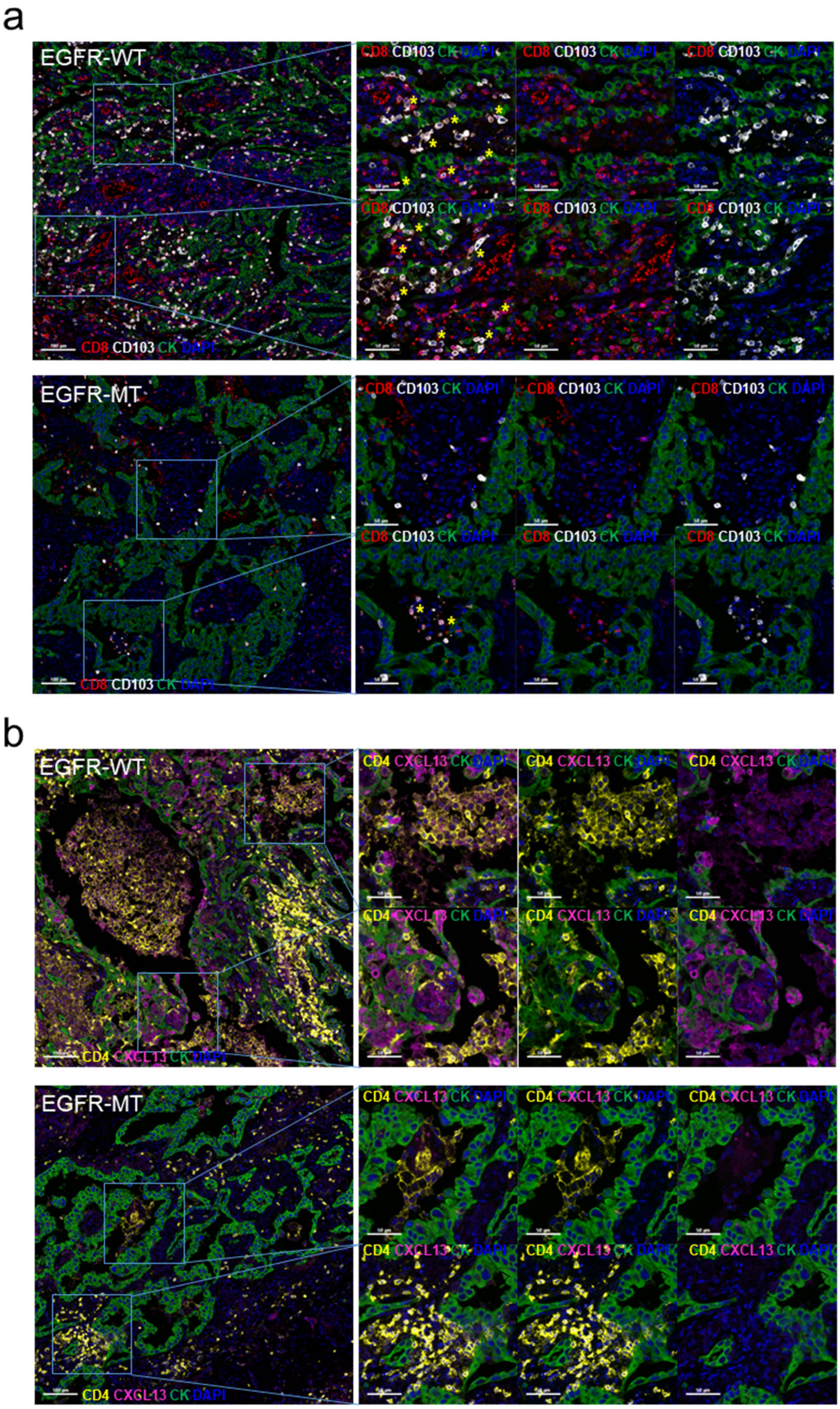
a
EGFR-WT
CD8 CD103 CK DAPI
EGFR-MT
CD8 CD103 CK DAPI
b
EGFR-WT
CD4 CXCL13 CK DAPI
EGFR-MT
CD4 CXCL13 CK DAPI

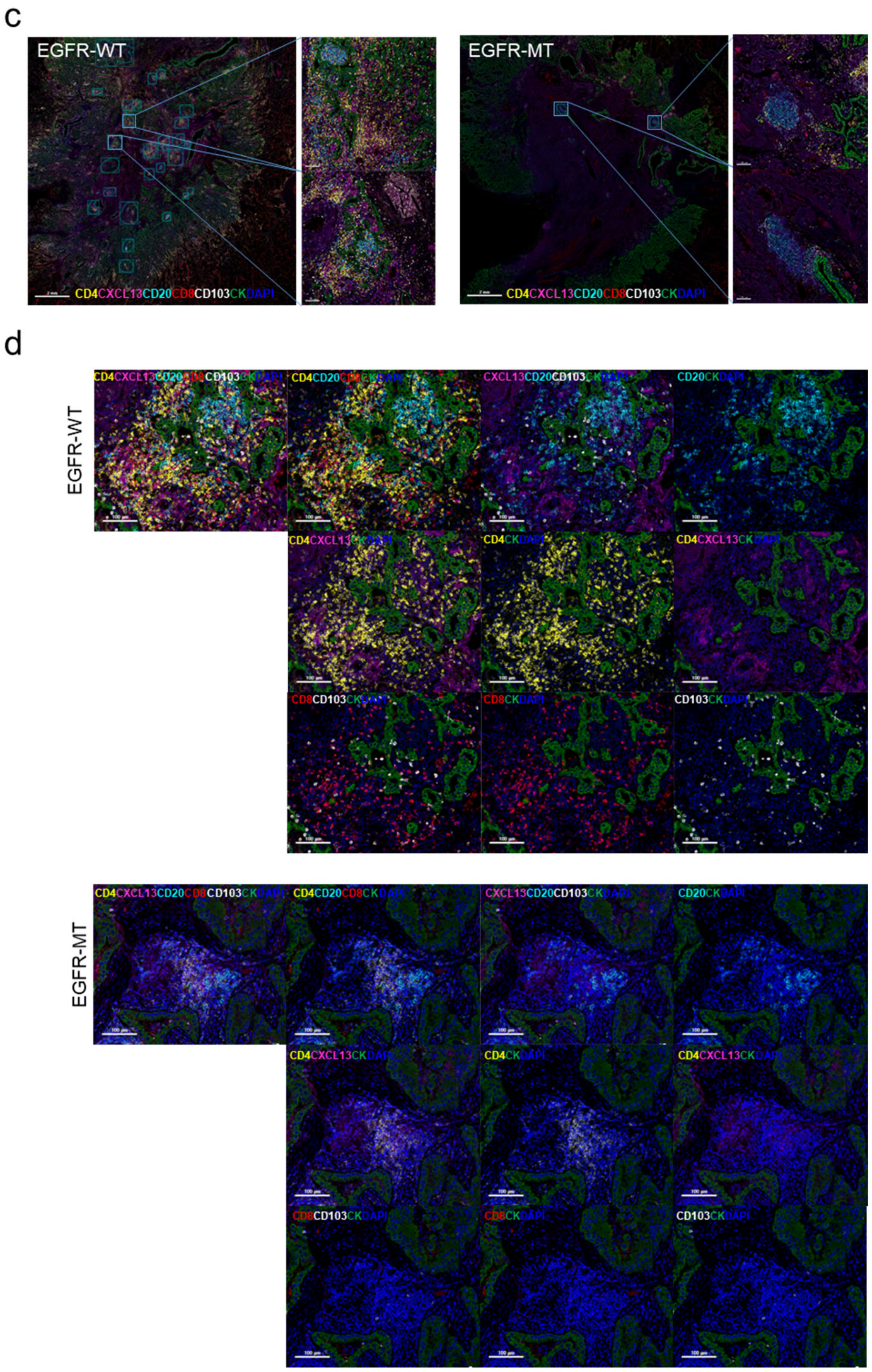

Figure S12. **Representative multiplexed IF of CD4, CXCL13, CD20, CD103, CD8 in tumor specimens from EGFR-WT and EGFR-MT in independent validation set.** Representative multiplexed IF of CD8 and CD103 in tumor specimens from EGFR-WT (n=9) (scale bar 100µm: 50µm (magnified)) (a) and EGFR-MT (n=10) (scale bar 100µm: 50µm (magnified)) (b) patients in whole slide. The dual positive $CD8^{+}CD103^{+}$ cells are marked by asterisk. (scale bar 2mm: 50µm (magnified)) (c) Representative multiplexed IF of TLS-like lesion in EGFR-WT (n=9) (left) and EGFR-MT (n=10) (right) tumors. Representative multiplexed IF (scale bar 100µm) of CD4, CXCL13, CD103, CD8, and CD20 in tumor specimens from EGFR-WT (n=9) (upper) and EGFR-MT (n=10) (lower) patients.

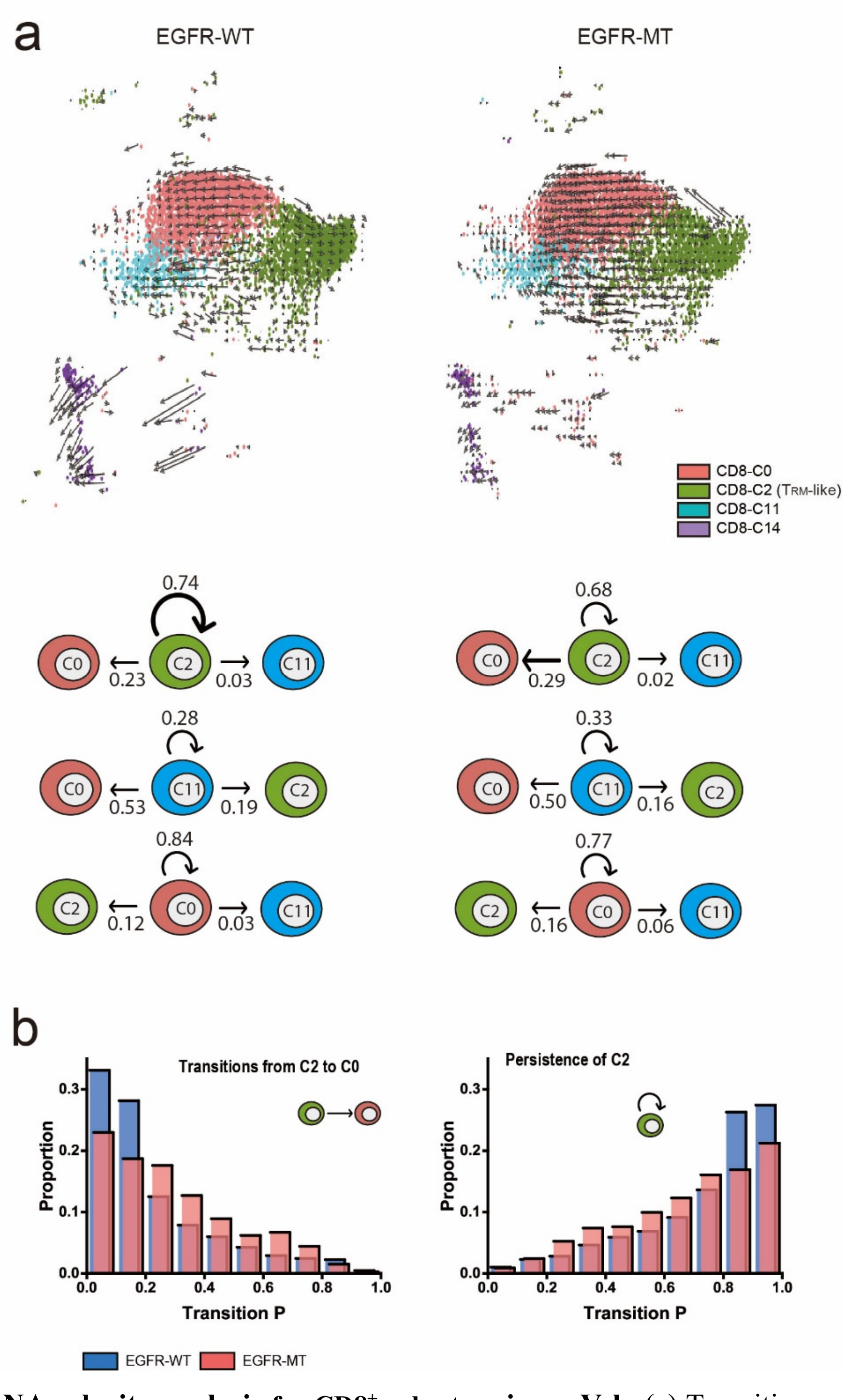

Figure S13. RNA velocity analysis for CD8$^+$ subsets using scVelo (a) Transition probability of cells is visualized on the predefined UMAP plots for CD8$^+$ T cells of EGFR-WT and EGFR-MT tumors. (b) Histograms for the distribution of transition probabilities from C2 subset to C0 subset (left panel) and those from C2 subset to C2 subset (right panel) in two different tumor groups. Source data are provided as a Source Data file.

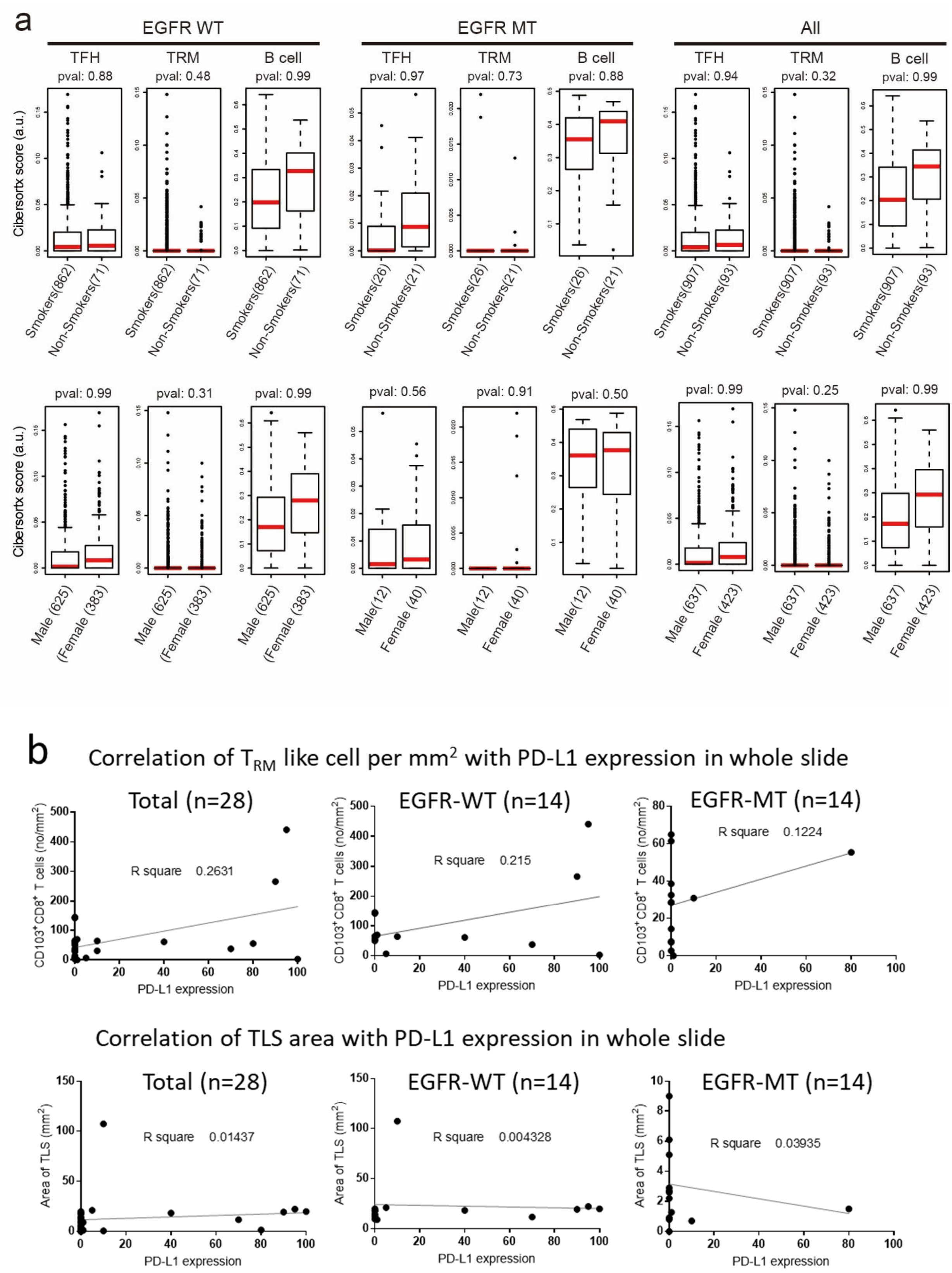

a
EGFR WT
EGFR MT
All
TFH
TRM
B cell
pval: 0.88
pval: 0.48
pval: 0.99
pval: 0.97
pval: 0.73
pval: 0.88
pval: 0.94
pval: 0.32
pval: 0.99
Cibersortx score (a.u.)
Smokers(862)
Non-Smokers(71)
Smokers(26)
Non-Smokers(21)
Smokers(907)
Non-Smokers(93)
pval: 0.99
pval: 0.31
pval: 0.99
pval: 0.56
pval: 0.91
pval: 0.50
pval: 0.99
pval: 0.25
pval: 0.99
Male (625)
(Female (383)
Male(12)
Female (40)
Male (637)
Female (423)
b
Correlation of T_RM like cell per mm^2 with PD-L1 expression in whole slide
Total (n=28)
EGFR-WT (n=14)
EGFR-MT (n=14)
R square 0.2631
R square 0.215
R square 0.1224
CD103+CD8+ T cells (no/mm2)
PD-L1 expression
Correlation of TLS area with PD-L1 expression in whole slide
R square 0.01437
R square 0.004328
R square 0.03935
Area of TLS (mm2)

Figure S14. Tests of confounding effect of smoking status, gender, and PD-L1 expression on association between EGFR mutation status and tumor microenvironment (TME) phenotypes. (a) We observed no significant decrease in the CIBERSORTx score for T_{RM}-like, Tfh-like, B cells in non-smokers or females from both EGFR-WT and EGFR-MT groups of the NSCLC tumors of TCGA cohort (one-tailed Mann-Whitney *U* test, sample sizes are indicated in parentheses), which indicates that smoking status and gender are not confounders of the observed association between EGFR mutation status and the TME phenotypes. In bar-and-whiskers plots, the central horizontal line in the box indicates the median score and the boundaries of the box indicate the first and third quartiles of the score distribution. Whiskers indicate the 10th and 90th percentiles, and circles indicate individual outliers. (b) We observed weak correlation of $CD103^{+}CD8^{+}$ T cells per mm^2 or tertiary lymphoid structure (TLS) area with PD-L1 expression in whole slide, which indicates that PD-L1 was not a confounder of the observed association between EGFR mutation status and the TME phenotypes. PD-L1 expression was quantified based on tumor proportion score (TPS). Source data are provided as a Source Data file.

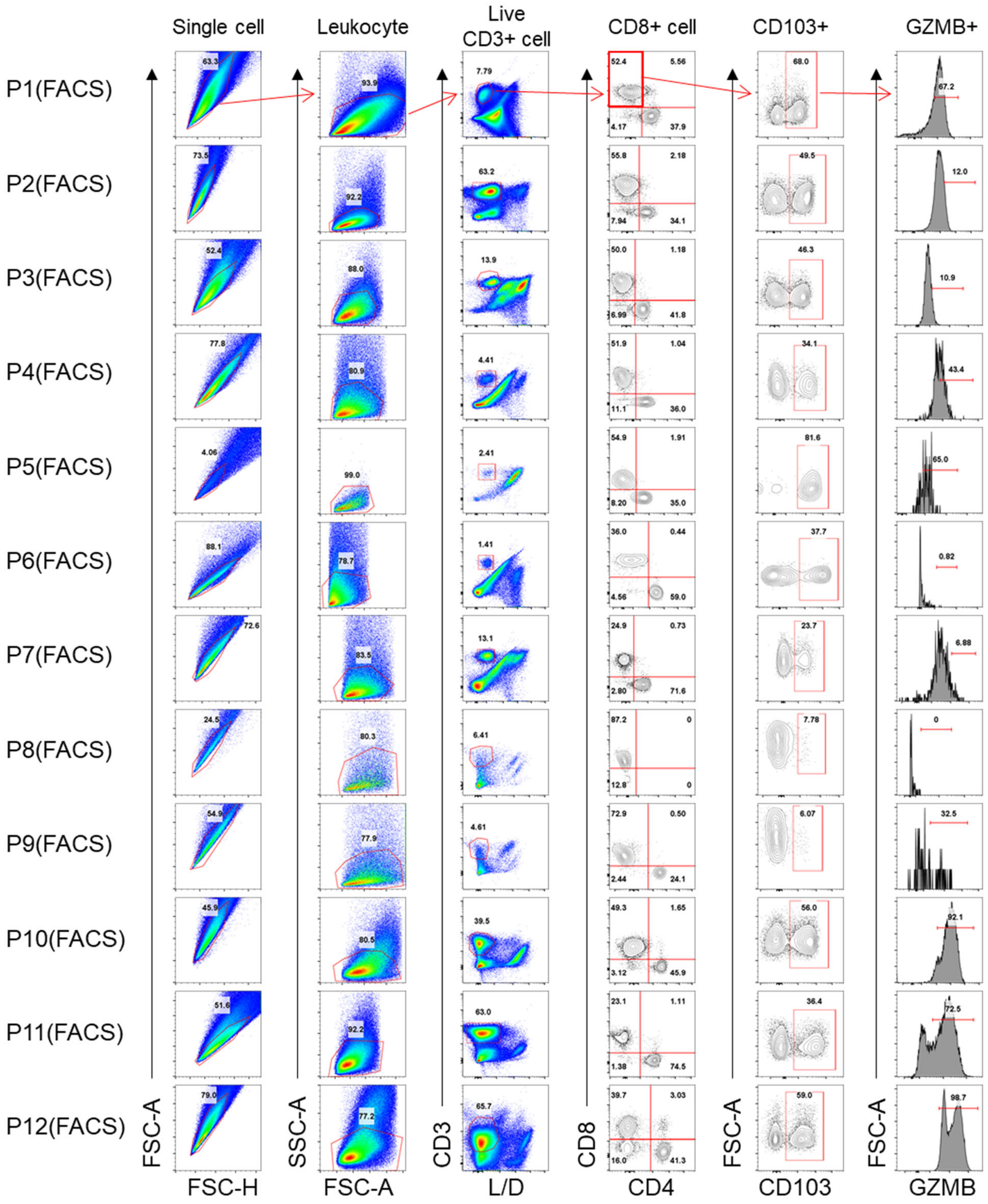
Single cell
Leukocyte
Live CD3+ cell
CD8+ cell
CD103+
GZMB+
P1(FACS)
P2(FACS)
P3(FACS)
P4(FACS)
P5(FACS)
P6(FACS)
P7(FACS)
P8(FACS)
P9(FACS)
P10(FACS)
P11(FACS)
P12(FACS)
FSC-A
FSC-H
SSC-A
FSC-A
CD3
L/D
CD8
CD4
FSC-A
CD103
FSC-A
GZMB

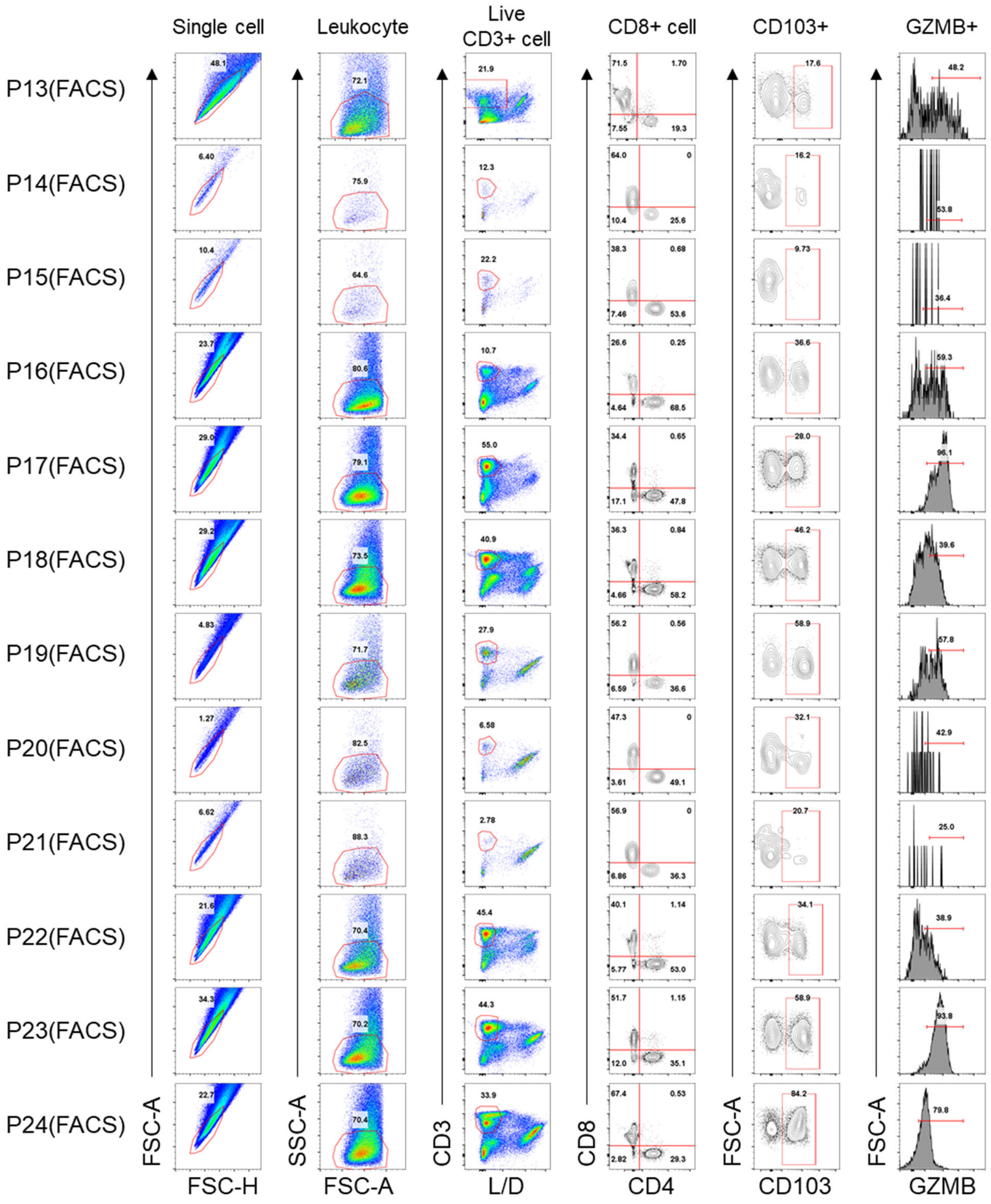
Single cell
Leukocyte
Live CD3+ cell
CD8+ cell
CD103+
GZMB+
P13(FACS)
P14(FACS)
P15(FACS)
P16(FACS)
P17(FACS)
P18(FACS)
P19(FACS)
P20(FACS)
P21(FACS)
P22(FACS)
P23(FACS)
P24(FACS)
FSC-A
FSC-H
SSC-A
FSC-A
CD3
L/D
CD8
CD4
FSC-A
CD103
FSC-A
GZMB

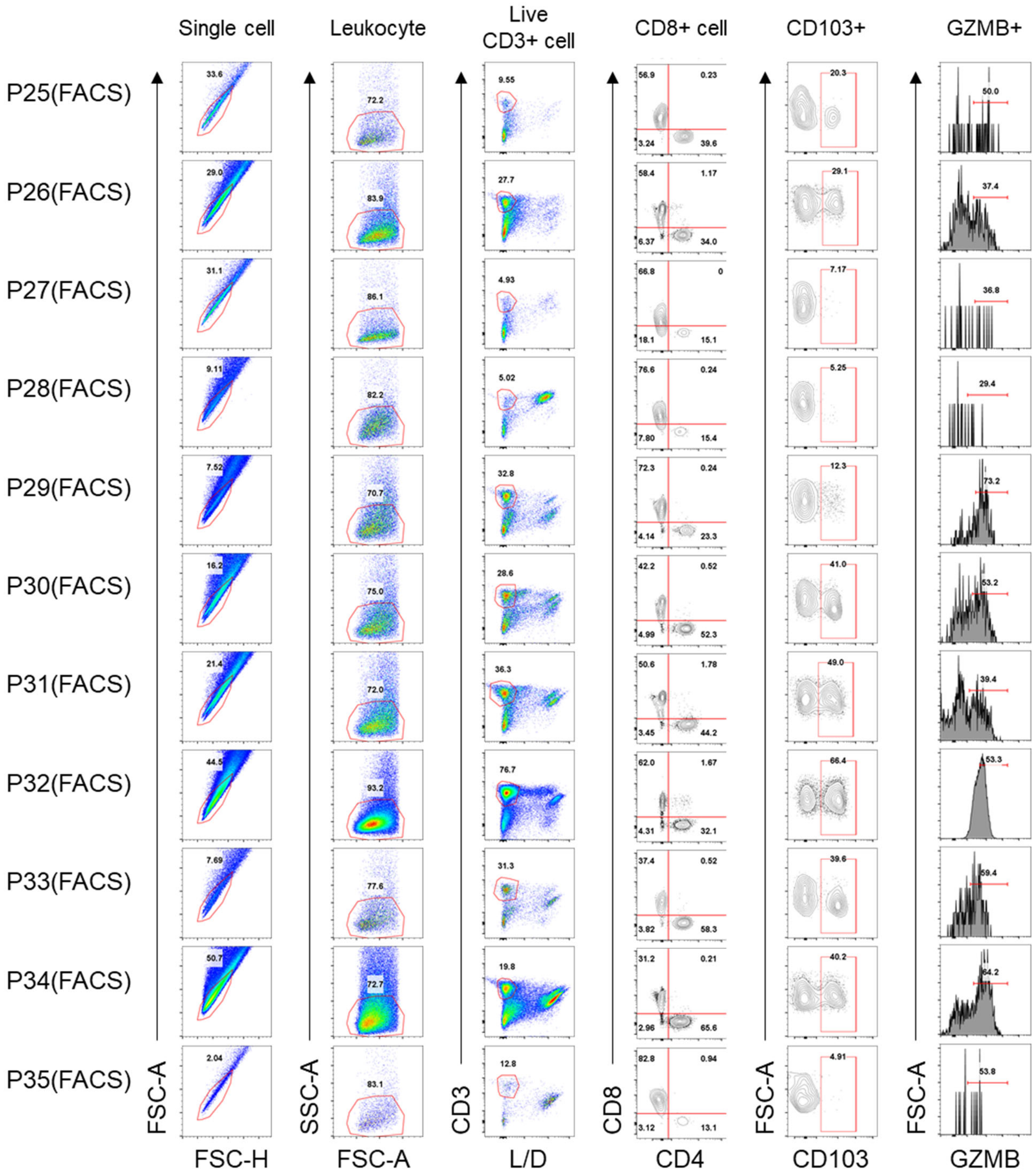

Figure S15. **Gating strategies used for CD103 and GZMB staining.** Gating the plot by forward scatter-area (FSC-A) against forward scatter-height (FSC-H) indicates single cells. Leukocytes are identified by forward scatter-area (FSC-A) against versus side scatter-area (SSC-A) plot. Live CD3 positive cells were separated by CD3 and LIVE/DEAD™ Stain Kit. CD8 positive cells were separated by gating on CD8 positive and CD4 negative.
