# Supplementary material for: Dysregulation of TFH-B-TRM lymphocyte cooperation is associated with unfavorable anti-PD-1 responses in EGFR-mutant lung cancer
Source: Nat Commun. 2021 Oct 18;12:6068. doi: 10.1038/s41467-021-26362-0 (PMC8523541; doi:10.1038/s41467-021-26362-0)
Supplement: Supplementary file 2 — Description of Additional Supplementary Files [file 41467_2021_26362_MOESM2_ESM.pdf]

Description of Additional Supplementary Files

File Name: Supplementary Data 1
Description: Information of clinical samples used in this study

File Name: Supplementary Data 2
Description: Cell counts information across samples and cell subsets

File Name: Supplementary Data 3
Description: Lists of differentially expressed genes

File Name: Supplementary Data 4
Description:

a. Cell counts of each subset per area in whole slide (n=9).
b. Total cell count of each subset in TLS-like lesions (n=9).
c. Cell counts of each subset per area in whole slide of independent set (n=19).
d. Total cell count of each subset in TLS-like lesions of independent set (n=19).

File Name: Supplementary Data 5
Description:

a. Baseline characateristics of paients with multiplex immunofluorescence as independent set.
b. Baseline characateristics of paients with flow cytometry

File Name: Supplementary Data 6
Description:

a. Transition probability of each subset in EGFR-WT and EGFR-MT
b. Transition probability of each cell in EGFR-WT
c. Transition probability of each cell in EGFR-MT

File Name: Supplementary Data 7
Description: Coexpression analysis based on Spearman correlation of NOTCH1 or RBPJ with genes in C2 (Trm-like cells) of EGFR-WT and EGFR-MT tumors (p-value is by two-sided test)

File Name: Supplementary Data 8
Description: Estimating relative abundance of Trm-like subset (C2) using CIBERSORTx

a. Signature matrix from CIBERSORTx analysis
b. Trm-like signature score for TCGA cohort: 52 EGFR-MT (L858R, S768I, L861Q, G719X, any exon19 del) and 964 EGFR-WT
c. Trm-like signature score for GSE128045 cohort: responder-5, non-responder-11
d. Trm-like signature score for GSE135222 cohort: responder-8, non-responder-19
