# Supplementary material for: Dysregulation of TFH-B-TRM lymphocyte cooperation is associated with unfavorable anti-PD-1 responses in EGFR-mutant lung cancer
Source: Nat Commun. 2021 Oct 18;12:6068. doi: 10.1038/s41467-021-26362-0 (PMC8523541; doi:10.1038/s41467-021-26362-0)
Supplement: Supplementary file 11 — Reporting Summary [file 41467_2021_26362_MOESM11_ESM.pdf]

nature research

Corresponding author(s): Insuk Lee
Hye Ryun Kim
Sang-Jun Ha
Seong Yong Park

Last updated by author(s): Jul 28, 2021

Reporting Summary

Nature Research wishes to improve the reproducibility of the work that we publish. This form provides structure for consistency and transparency in reporting. For further information on Nature Research policies, see our Editorial Policies and the Editorial Policy Checklist.

Statistics

For all statistical analyses, confirm that the following items are present in the figure legend, table legend, main text, or Methods section.

n/a	Confirmed	
☐	☒	The exact sample size (*n*) for each experimental group/condition, given as a discrete number and unit of measurement
☐	☒	A statement on whether measurements were taken from distinct samples or whether the same sample was measured repeatedly
☐	☒	The statistical test(s) used AND whether they are one- or two-sided<br>*Only common tests should be described solely by name; describe more complex techniques in the Methods section.*
☒	☐	A description of all covariates tested
☐	☒	A description of any assumptions or corrections, such as tests of normality and adjustment for multiple comparisons
☐	☒	A full description of the statistical parameters including central tendency (e.g. means) or other basic estimates (e.g. regression coefficient) AND variation (e.g. standard deviation) or associated estimates of uncertainty (e.g. confidence intervals)
☐	☒	For null hypothesis testing, the test statistic (e.g. *F*, *t*, *r*) with confidence intervals, effect sizes, degrees of freedom and *P* value noted<br>*Give P values as exact values whenever suitable.*
☒	☐	For Bayesian analysis, information on the choice of priors and Markov chain Monte Carlo settings
☐	☒	For hierarchical and complex designs, identification of the appropriate level for tests and full reporting of outcomes
☐	☒	Estimates of effect sizes (e.g. Cohen's *d*, Pearson's *r*), indicating how they were calculated

Our web collection on statistics for biologists contains articles on many of the points above.

Software and code

Policy information about availability of computer code

Data collection	No software was used for data collection. All scRNA-seq in the study were generated by the research group.
Data analysis	- Single-cell RNA sequencing data analysis<br>Python (version 3.6)<br>Cell Ranger (version 2.1.1)<br>Seurat (version 3.0)<br>Velocyto (version 0.17.16)<br>scVelo (version 0.2.3)<br>MetaCell (version 0.3.41)<br>CIBERSORTx (online version)<br>CellPhoneDB (version 2.0.0)<br><br>- Image data analysis<br>InForm (version 2.2)<br>TIBCO Spotfire (version 7.11.1)<br><br>- Flow cytometry data analysis<br>FlowJo (version 10.6.2)

For manuscripts utilizing custom algorithms or software that are central to the research but not yet described in published literature, software must be made available to editors and reviewers. We strongly encourage code deposition in a community repository (e.g. GitHub). See the Nature Research guidelines for submitting code & software for further information.

Data

Policy information about availability of data

All manuscripts must include a data availability statement. This statement should provide the following information, where applicable:

- Accession codes, unique identifiers, or web links for publicly available datasets
- A list of figures that have associated raw data
- A description of any restrictions on data availability

The single-cell RNA sequencing data generated in this study have been deposited in the Gene Expression Omnibus database (GSE144945, https://www.ncbi.nlm.nih.gov/geo/query/acc.cgi?acc=GSE144945). TCGA cohort data set were obtained from [https://gdac.broadinstitute.org]. Bulk RNA-seq data for two public cohorts of patients with lung cancer treated with anti-PD-1 immunotherapy were obtained from GSE126045 [https://www.ncbi.nlm.nih.gov/geo/query/acc.cgi?acc=GSE126045] and GSE135222 [https://www.ncbi.nlm.nih.gov/geo/query/acc.cgi?acc=GSE135222]. The remaining data are available within the Article, Supplementary Information or Source Data file.

Field-specific reporting

Please select the one below that is the best fit for your research. If you are not sure, read the appropriate sections before making your selection.

- [x] Life sciences
- [ ] Behavioural & social sciences
- [ ] Ecological, evolutionary & environmental sciences

For a reference copy of the document with all sections, see nature.com/documents/nr-reporting-summary-flat.pdf

Life sciences study design

All studies must disclose on these points even when the disclosure is negative.

Sample size	Patients diagnosed with NSCLC and who underwent surgical resection at Severance Hospital (Seoul, Republic of Korea), between June 2018 and August 2018, were prospectively enrolled. We consecutively enrolled five patients with EGFR-MT and five patients with EGFR-WT lung cancer. Total 10 samples (5 per each group) were used for the analysis. For validation in independent set, additional 35 NSCLC patients with EGFR-WT (n = 16) or EGFR-MT (n = 19) were included. Sample size for each experiment is described in the main manuscript and is based on the availability of adequate patients' specimens. No sample size calculations were performed prior to the study.
Data exclusions	We applied pre-established quality assessment and data excluded criteria on raw gene matrix data for the single cells with outlier behaviors in the number of detected genes and the number of mitochondrial genes of the total counts.
Replication	As the expected cellular numbers were collected from the scRNA-seq data based on the 10x Genomics platform, the scRNA-seq for each patients was not repeatedly performed.
Randomization	No randomization was used in this study as no experimental treatments were involved.
Blinding	Not applicable because this is a descriptive study and no interventions were tested.

Reporting for specific materials, systems and methods

We require information from authors about some types of materials, experimental systems and methods used in many studies. Here, indicate whether each material, system or method listed is relevant to your study. If you are not sure if a list item applies to your research, read the appropriate section before selecting a response.

Materials & experimental systems

n/a	Involved in the study	
[ ]	[x]	Antibodies
[x]	[ ]	Eukaryotic cell lines
[x]	[ ]	Palaeontology and archaeology
[x]	[ ]	Animals and other organisms
[ ]	[x]	Human research participants
[x]	[ ]	Clinical data
[x]	[ ]	Dual use research of concern

Methods

n/a	Involved in the study	
[x]	[ ]	ChIP-seq
[ ]	[x]	Flow cytometry
[x]	[ ]	MRI-based neuroimaging

Antibodies

Antibodies used	The antibodies used for Multiplex immunofluorescence listed below.<br>CD103 (ab129202, Abcam, Cambridge, UK) 1:500, GZMB (262A-15, Cell Marque, Rocklin, CA, USA) 1:50, CD4 (ab133616, Abcam, Cambridge, UK) 1:200, CD8 (MCA1817, Bio-Rad, Hercules, CA, USA) 1:300, CXCL13 (PA5-47035, Invitrogen) 1:40, CXCR5 (72172S, CST, dilution 1:200) 1:200, CD20 (ab9475, Abcam, Cambridge, UK) 1:100

The antibodies used for Flow cytometry listed below:
CD8 (RPA-T8, cat# 301048, Biolegend, San Diego, CA, USA, 1: 50), CD3 (SK7, cat# 344808, Biolegend, San Diego, CA, USA, 1: 100), PD-1 (EH12.2H7, cat# 344808, Biolegend, San Diego, CA, USA, 1:20), CD103 (Ber-ACT8, cat# 350230, Biolegend, San Diego, CA, USA, 1:20), CD39 (A1, cat# 328210, Biolegend, San Diego, CA, USA, 1:20), GZMB (QA16A02, cat# 372214, Biolegend, San Diego, CA, USA, 1:50), CD4 (RPA-T4, cat# 560837, BD Biosciences, San Diego, CA, USA, 1:50), and LIVE/DEAD Fixable Near-IR Dead Cell Stain kit (Invitrogen, L34973, Waltham, Massachusetts, USA, 1:100). CD45 (clone 2D1, Biolegend, San Diego, CA, USA, 1:100).

Validation

The primary antibodies used in this study are widely used and well validated. The mentioned antibodies are tested by immunofluorescent staining with flow cytometric analysis by the manufacturer. The following information is available through the manufacturers' websites:
The antibodies used for Multiplex immunofluorescence listed below.
CD103 (ab129202): https://www.abcam.com/cd103-antibody-epr41662-ab129202.html
GZMB (262A-15): https://www.cellmarque.com/antibodies/CM/2026/Granzyme-B_polyclonal
CD4 (ab133616): https://www.abcam.com/cd4-antibody-epr6855-ab133616.html
CD8 (MCA1817): https://www.bio-rad-antibodies.com/monoclonal/human-cd8-antibody-4b11-mca1817.html?f=s%2Fn
CXCL13 (PA5-47035): https://www.thermofisher.com/antibody/product/CXCL13-Antibody-Polyclonal/PA5-47035
CXCR5 (72172S): https://www.cellsignal.com/products/primary-antibodies/cxcr5-d6l3c-rabbit-mab-ihc-specific/72172
CD20 (ab9475): https://www.abcam.com/cd20-antibody-l26-ab9475.html
The antibodies used for Flow cytometry listed below:
CD8 (RPA-T8, Biolegend, cat# 301048) https://www.biolegend.com/en-us/search-results/brilliant-violet-510-anti-human-cd8a-antibody-8000?GroupID=BLG5903
CD3 (SK7, Biolegend, cat# 344808): https://www.biolegend.com/en-us/search-results/percp-cyanine5-5-anti-human-cd3-antibody-6932?GroupID=BLG7568
PD-1 (EH12.2H7, Biolegend, cat# 344808): https://www.biolegend.com/en-us/search-results/pe-anti-human-cd279-pd-1-antibody-4412?GroupID=BLG5466
CD103 (Ber-ACT8, Biolegend, cat# 350230): https://www.biolegend.com/en-us/products/brilliant-violet-785-anti-human-cd103-integrin-alpha-e-antibody-16015?GroupID=BLG15664
CD39 (A1, Biolegend, cat# 328210): https://www.biolegend.com/fr-ch/products/apc-anti-human-cd39-antibody-6275
GZMB (QA16A02, Biolegend, cat# 372214): https://www.biolegend.com/en-ie/products/pe-cyanine7-anti-humanmouse-granzyme-b-recombinant-antibody-15582?GroupID=GROUP28
CD4 (RPA-T4, BD Biosciences, cat# 560837): https://www.bdbiosciences.com/en-eu/products/reagents/flow-cytometry-reagents/research-reagents/single-color-antibodies-ruo/apc-h7-mouse-anti-human-cd4.560837
LIVE/DEAD Fixable Near-IR Dead Cell Stain kit (Invitrogen): https://www.thermofisher.com/order/catalog/product/L34973#/L34973
CD45 (clone 2D1, Biolegend, San Diego, CA, USA, 1:100).

Human research participants

Policy information about studies involving human research participants

Population characteristics

In discovery set for scRNA-seq, a total of 10 patients (age 44-75) diagnosed with NSCLC and undergoing surgery were recruited. Six patients were in pathological stage I, two in stage II, and two in stage III. In validation cohort for flow cytometry, 35 NSCLC patients (age 41-86) undergoing surgery were enrolled. 21 patients were in stage I, four in stage II, nine in stage III, and 1 in stage IV. In validation cohort for multiplex IF, 19 patients (age 45-83) were included. 3 patients were in stage I, 13 in stage II, and 3 in stage III. No patient had undergone chemotherapy or radiation therapy before surgical resection.

Recruitment

Patients diagnosed with NSCLC and who underwent surgical resection at Severance Hospital (Seoul, Republic of Korea), between June 2018 and August 2018, were consented to collect specimens. Recruitment was focused on patients presenting with operable primary tumors.

Ethics oversight

NSCLC patients undergoing surgical resection were enrolled in accordance with an approved Severance Hospital Institutional Review Board (IRB No 4-2018-1210), with all patients providing informed consent.

Note that full information on the approval of the study protocol must also be provided in the manuscript.

Flow Cytometry

Plots

Confirm that:

- [x] The axis labels state the marker and fluorochrome used (e.g. CD4-FITC).
- [x] The axis scales are clearly visible. Include numbers along axes only for bottom left plot of group (a 'group' is an analysis of identical markers).
- [x] All plots are contour plots with outliers or pseudocolor plots.
- [x] A numerical value for number of cells or percentage (with statistics) is provided.

Methodology

Sample preparation	We collected fresh tumor tissues on the day of the surgery, which was mechanically and enzymatically dissociated using a gentleMACS dissociator (Miltenyi Biotec, Gladbach Bergisch, Germany, Cat#130-093-235) and the Human Tumor Dissociation Kit (Miltenyi Biotec, Cat#130-095-929) following the manufacturer's instructions. After incubation for 1 h at 37 ℃, the resuspended samples were filtered through a 70-µm MACS SmartStrainer (Miltenyi Biotec, Cat# 130-098-462) into RPMI-1640 medium (Corning, Inc., Corning, NY, USA) supplemented with 10% fetal bovine serum (Biowest, Riverside, MO, USA) and centrifuged at 300 ×g for 10 min. The pellet was suspended in PBS containing 2% fetal bovine serum. For fluorescence-activated cell sorting (FACS) analysis, single-cell suspensions were stained for 20 min at 4 ℃ with the following fluorescent-dye-conjugated antibodies: LIVE/DEAD™ Fixable Red Dead Cell Stain Kit (Invitrogen, Carlsbad, CA, USA, Cat#L23102) and CD45 (Biolegend, San Diego, CA, USA, clone 2D1).
Instrument	Flow cytometry was performed using CytoFLEX (Beckman Coulter, Brea, CA, USA).
Software	Data were analyzed using FlowJo software (Tree Star, Ashland, OR, USA).
Cell population abundance	Post-sort purity was analysed for samples with more than 5000 target cells collected. The purity of the sorted cells was greater than 97%.
Gating strategy	The samples were sorted using a FACS Aria III sorter (BD Biosciences, Franklin Lakes, NJ, USA) into live and single CD45+ cells. The sorted cell population was analyzed to ensure successful cell sorting, as shown in Fig. S1. Cell viability was assessed by the Luna FL Fluorescence Cell Counter (Logos Biosystems, Anyang, Korea, Cat#L20001) and confirmed to be 75–98% in all samples.

☒ Tick this box to confirm that a figure exemplifying the gating strategy is provided in the Supplementary Information.
